# Revelation of Potent Epitopes Present in Unannotated ORF Antigens of SARS-CoV-2 for Epitope-Based Polyvalent Vaccine Design Using Immunoinformatics Approach

**DOI:** 10.3389/fimmu.2021.692937

**Published:** 2021-08-23

**Authors:** Patil Pranita Uttamrao, Chakkarai Sathyaseelan, L. Ponoop Prasad Patro, Thenmalarchelvi Rathinavelan

**Affiliations:** Department of Biotechnology, Indian Institute of Technology Hyderabad, Kandi, India

**Keywords:** uORFs, SARS-CoV-2, ORF9b, T-cell epitopes, B-cell epitopes, epitope-based polyvalent vaccine, Canonical protein epitopes, HLA I supergroups

## Abstract

Severe acute respiratory syndrome coronavirus 2 (SARS-CoV-2) kills thousands of people worldwide every day, thus necessitating rapid development of countermeasures. Immunoinformatics analyses carried out here in search of immunodominant regions in recently identified SARS-CoV-2 unannotated open reading frames (uORFs) have identified eight linear B-cell, one conformational B-cell, 10 CD4+ T-cell, and 12 CD8+ T-cell promising epitopes. Among them, ORF9b B-cell and T-cell epitopes are the most promising followed by M.ext and ORF3c epitopes. ORF9b_40-48_ (CD8+ T-cell epitope) is found to be highly immunogenic and antigenic with the highest allele coverage. Furthermore, it has overlap with four potent CD4+ T-cell epitopes. Structure-based B-cell epitope prediction has identified ORF9b_61-68_ to be immunodominant, which partially overlaps with one of the linear B-cell epitopes (ORF9b_65-69_). ORF3c CD4+ T-cell epitopes (ORF3c_2-16_, ORF3c_3-17_, and ORF3c_4-18_) and linear B-cell epitope (ORF3c_14-22_) have also been identified as the candidate epitopes. Similarly, M.ext and 7a.iORF1 (overlap with M and ORF7a) proteins have promising immunogenic regions. By considering the level of antigen expression, four ORF9b and five M.ext epitopes are finally shortlisted as potent epitopes. Mutation analysis has further revealed that the shortlisted potent uORF epitopes are resistant to recurrent mutations. Additionally, four N-protein (expressed by canonical ORF) epitopes are found to be potent. Thus, SARS-CoV-2 uORF B-cell and T-cell epitopes identified here along with canonical ORF epitopes may aid in the design of a promising epitope-based polyvalent vaccine (when connected through appropriate linkers) against SARS-CoV-2. Such a vaccine can act as a bulwark against SARS-CoV-2, especially in the scenario of emergence of variants with recurring mutations in the spike protein.

## Introduction

Even 18 months after the official declaration of the SARS-CoV-2 pandemic by the World Health Organization (https://www.who.int/), the world is losing thousands of lives, and nearly half a million people around the globe are being infected by the virus every day (https://www.worldometers.info/coronavirus/). Although spike glycoprotein-based vaccines have been developed in a fast-track mode to combat SARS-CoV-2 (1–3), the viral evolution with mutations (4, 5) in spike protein and the associated enhanced pathogenicity, transmissibility, and immune escape are of major concerns (6). Indeed, there are reports (7, 8) about the reduced efficacy of the vaccines against the new variants (4). Reports indicate that the number of mutations in the spike protein has increased to 1.4-fold in a time span of 6 months (9, 10). This is indicative of challenges in using the existing spike protein antigen-based vaccines (11) when new variants emerge.

The efficient approach for vaccine development is the multiepitope-based vaccine, which uses short synthetic amino acid stretches that are present in the antigenic protein(s) and are capable of inducing a broad immune response (12). Experimental investigations in recent times have revealed immunodominant epitopes present in the canonical proteins of SARS-CoV-2 (13–25). Several immunoinformatics approaches, which are cost-effective and time-saving compared to the traditional methods, have also been used in this direction to identify potential epitopes in the canonical proteins of SARS-CoV-2 (26–35). Using comparative genomics and ribosome-profiling techniques, a recent experimental study has confirmed the translation of 23 additional unannotated open reading frame (uORF) proteins along with the proteins expressed by canonical ORFs (36). Despite being expressed in equivalence to the canonical ORF proteins and having functional and regulatory roles, uORFs are being neglected while analyzing the SARS-CoV-2 proteome dynamics. Nonetheless, to the best of our knowledge, there is no systematic investigation carried out to identify immunodominant regions in the uORF proteins of SARS-CoV-2.

**Figure 1:**
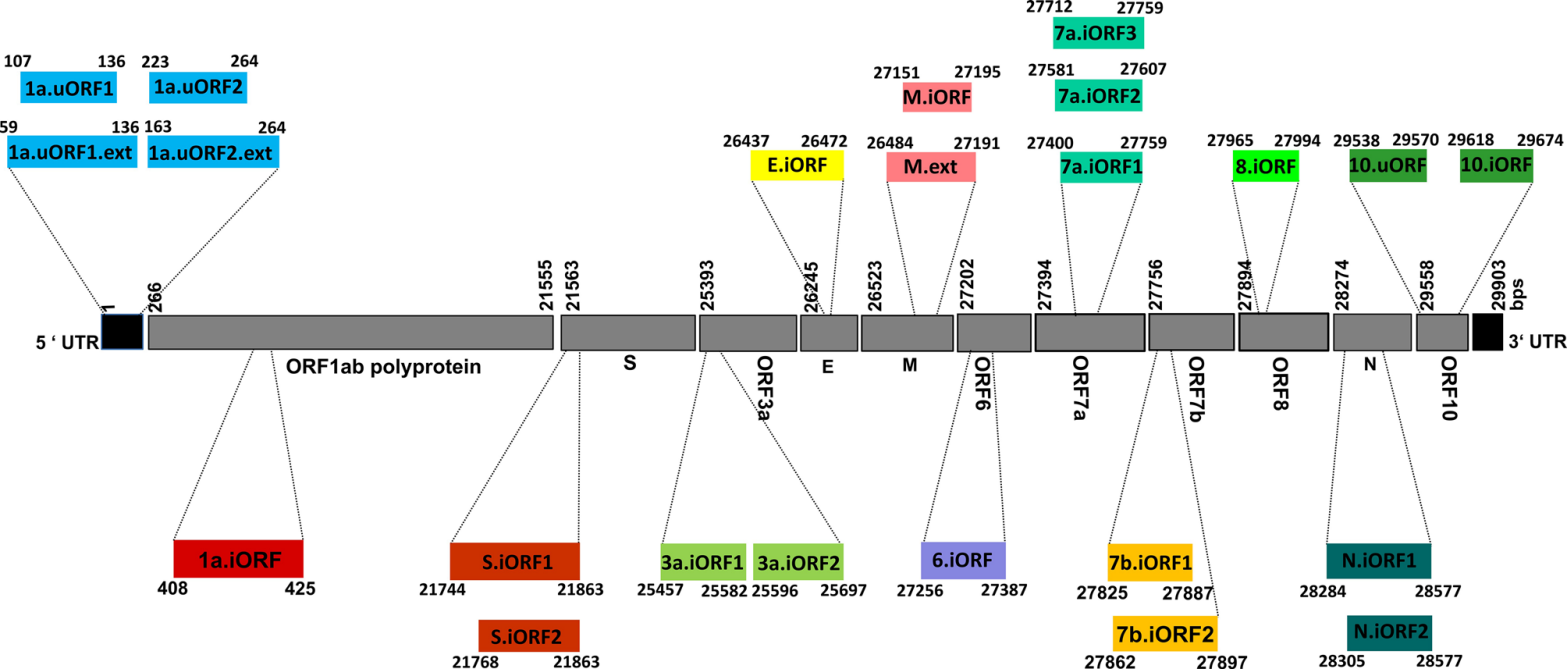
Schematic representation illustrating the genomic architecture of severe acute respiratory syndrome coronavirus 2 (SARS-CoV-2) unannotated open reading frames (uORFs). The numerical values given alongside the individual coding regions correspond to the nucleotide positions defined with respect to the whole genome sequence.

Thus, an immunoinformatics approach has been employed here to identify the potential T-cell and B-cell epitopes present in the antigens expressed by SARS-CoV-2 uORFs (**Figure 1** and **Table S1**). Although the SARS-CoV-2 uORFs express 23 proteins (36), nine of them are short polypeptide chains (*viz.*, less than 15 amino acids length) (36). Thus, 14 uORF proteins are considered in the current investigation to identify the potential B-cell linear epitopes and CD4+ T-cell [major histocompatibility complex (MHC) II/human leukocyte antigen (HLA) II] epitopes. Subsequently, 21 uORF proteins (with length of ≥9 amino acids) have been considered for CD8+ T-cell (MHC I/HLA I) epitope prediction. Furthermore, variations in these uORFs have also been analyzed by considering the 775,392 SARS-CoV-2 whole genome sequences deposited to GISAID until April 30, 2021. The results reveal several high, moderate, and low recurrent mutations located in the predicted promiscuous epitopes. However, promiscuous ORF9b epitopes are found to be resistant to mutations as well as immunogenic. It is noteworthy that ORF9b plays a role in inhibiting the host innate immune response (37), and it also has a good level of expression. Thus, the potent B-cell and T-cell epitopes identified in ORF9b make it a promising vaccine candidate. Similarly, N and M.ext/M proteins (**Figure 1** and **Table S1**) also possess potent epitopes. Finally, a vaccine construct has been proposed here by considering the potent epitopes of ORF9b, N and M.ext/M proteins.

## Methods

**Figure 2:**
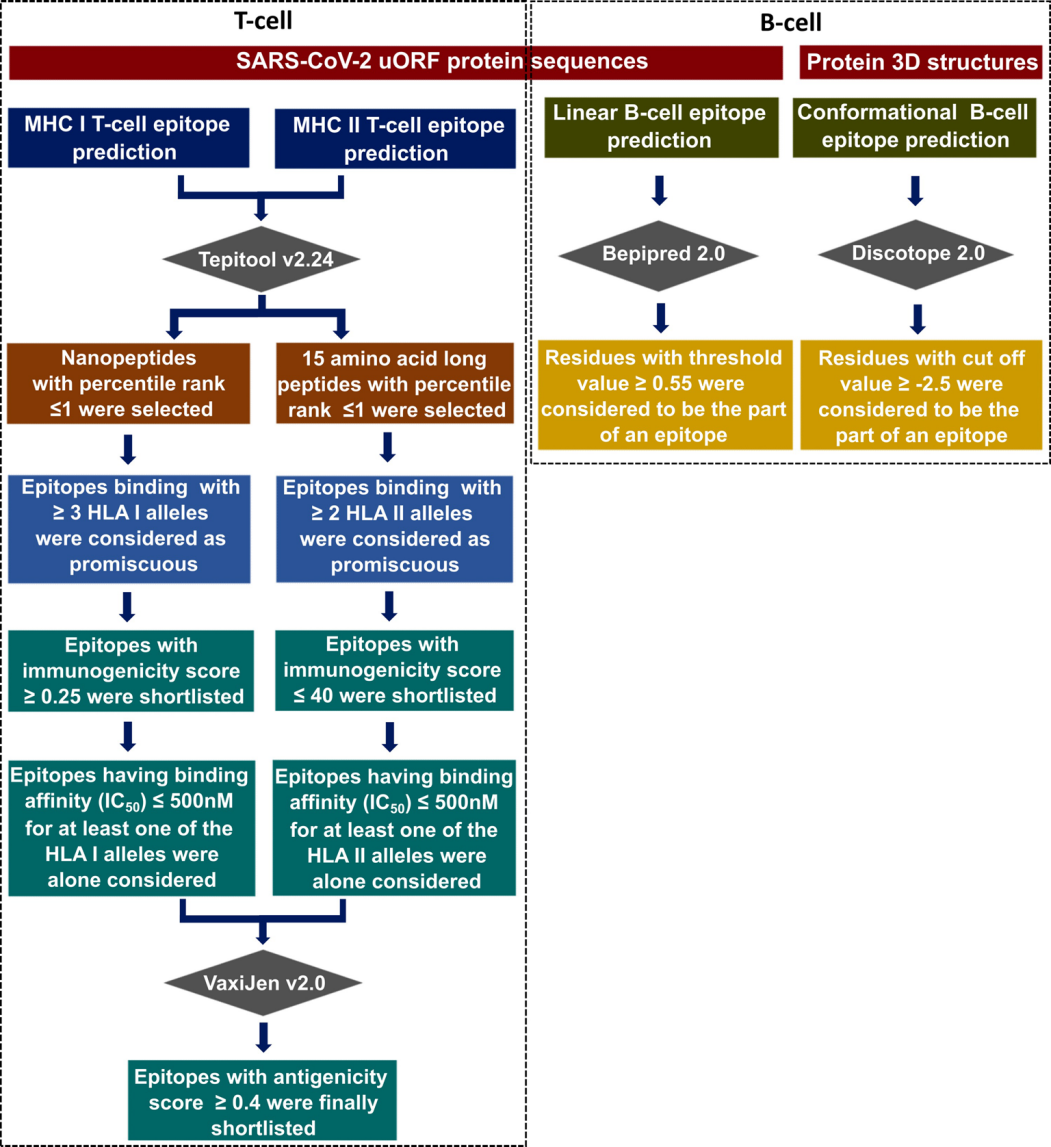
Flowchart illustrating the immunoinformatics protocol used in this study to identify the potential CD8+ T-cell [major histocompatibility complex (MHC) I/human leukocyte antigen (HLA) I] epitopes, CD4+ T-cell (MHC II/HLA II) epitopes, B-cell linear, and B-cell conformational epitopes corresponding to severe acute respiratory syndrome coronavirus 2 (SARS-CoV-2) canonical and unannotated open reading frames (uORFs) protein antigens.

The published SARS-CoV-2 uORF sequences (36) were used as the reference to predict the T-cell and B-cell epitopes present in the uORF proteins. For the mutation analyses, the coding regions corresponding to the uORFs were translated to amino acid sequences using the in-house scripts. **Figure 2** describes the epitope prediction methodology.

### T-Cell Epitope Prediction

CD8+ T-cell (MHC I/HLA I) epitope prediction was done against the 78 HLA class I alleles [HLA-A, HLA-B, HLA-C, HLA-E, and HLA-G (given in **Table S2**)] using the TepiTool resource from IEDB web tool (38). The epitopes (9-mer peptides) having the percentile rank ≤1 (estimated using the combination of ANN, SMM, CombLib, and NetMHCpan EL methods) were shortlisted for further analysis.

Parallelly, CD4+ T-cell (MHC II/HLA II) epitope prediction was done using the combination of NN-align, SMM-align, CombLib, Sturniolo, and NetMHCII-pan methods against the 27 HLA II alleles [HLA-DR, HLA-DP, and HLA-DQ (**Table S2**)]. The epitopes (15-mer) having the percentile rank ≤1 were shortlisted.

From the pool of epitopes, promiscuous epitopes were chosen based on their ability to bind multiple alleles, *viz.*, ≥3 and ≥2 for CD8+ and CD4+ T-cell epitopes, respectively. Furthermore, immunogenicity of the CD8+ T-cell epitopes was predicted using the MHC I immunogenicity analysis resource of IEDB (39), and the epitopes with an immunogenicity score ≥0.25 were shortlisted. Similarly, immunogenicity for CD4+ T-cell epitopes was checked using the CD4+ T-cell immunogenicity prediction tool of IEDB (40, 41), and epitopes having a combined immunogenicity score of ≤40 were shortlisted for further analysis.

### Characterization and Profiling of Predicted T-Cell Epitopes

Among the shortlisted epitopes, the ones that are having IC_50_ ≤500 nM (42) for at least one of its corresponding HLA alleles were alone considered to have good binding affinity. Antigenicity scores for both HLA I and HLA II epitopes (which fulfill the abovementioned IC_50_ criterion) were predicted using VaxiJen v2.0 (http://www.ddg-pharmfac.net/vaxijen/VaxiJen/VaxiJen.html) (43). Subsequently, the epitopes with a threshold of 0.4 antigenicity score were alone considered. Furthermore, the worldwide coverage of individual shortlisted epitopes was predicted using the population coverage analysis tool (44). For this, only 78 HLA class I alleles were considered, as they have more than 1% population frequency. The epitopes binding with HLA class I supergroups [seven supergroups (45)] and supertypes [10 supertypes (46)] were also analyzed to confirm the population coverage of the promiscuous epitopes.

### Linear and Conformational B-Cell Epitope Prediction

For linear (continuous) B-cell epitope prediction, protein sequences were examined for putative B-cell epitopes using the Bepipred 2.0 server (47) by applying the threshold value of ≥0.55 (corresponding to 80% specificity). For the conformational (discontinuous) B-cell epitope prediction, the Discotope 2.0 server (48) (cutoff ≥-2.5, corresponding to 80% specificity) was employed. Predicted epitopes were then projected onto the 3D structure(s) of protein antigen(s) using the PyMOL suite (49).

### Determination of SARS-CoV-2 uORF Sequence Conservation

To further evaluate the epitopes based on their sequence conservation, 775,392 SARS-CoV-2 whole genome sequences (deposited in the GISAID on or before April 30, 2021) were subjected to nucleotide and amino acid mutation analyses. For this, the gene sequences corresponding to the uORFs were translated using the reference sequences (36) with the help of in-house scripts. The amino acid mutation analyses were done as discussed elsewhere (9, 10). The mutations were categorized as highly recurring (HR, occurring with ≥10% percentage frequency), moderately recurring (MR, occurring with 1%–10% percentage frequency), and low recurring (LR, occurring below 1% percentage frequency but should have occurred at least three times) based on their recurrence in the 775,392 viral proteomes.

## Results

IEDB, a widely used resource to identify epitopes (www.iedb.org) (50), is employed in this study to predict the conformational B-cell (≥5-mer peptide), linear B-cell (5–30-mer peptide), CD8+ T-cell (HLA I) (9-mer peptide), and CD4+ T-cell (HLA II) (15-mer peptide) epitopes present in the SARS-CoV-2 uORF proteins. Based on the stringent criteria described in the *Methods* section (**Figure 2**), the potent CD8+ T-cell and CD4+ T-cell epitopes are shortlisted from the pool of predicted epitopes. A CD8+ or CD4+ T-cell epitope is considered a potent epitope only when it fulfills the criteria of promiscuity, immunogenicity, antigenicity, and binding affinity. For instance, although a CD8+ T-cell epitope binds with more than three HLA I alleles and has immunogenicity and antigenicity scores above 0.25 and 0.4, respectively, it is not considered a potent epitope if the IC_50_ value is not less than 500 nM for at least one of its HLA I-binding partners.

By applying the above criteria (**Figure 2**), one conformational B-cell, 10 linear B-cell, 13 CD8+ T-cell (HLA I), and 17 CD4+ T-cell (HLA II) epitopes are shortlisted (**Tables 1** **–** **3**). Very interestingly, five of the ORF9b T-cell epitopes have partial overlap with the epitopes found in the SARS-CoV Tor2 strain (Source: IEDB) (www.iedb.org).

**Table 1:** Promiscuous B-cell epitopes from severe acute respiratory syndrome coronavirus 2 (SARS-CoV-2) unannotated open reading frame (uORF) proteins are listed along with the highly recurring (HR) or moderately recurring (MR) mutations, if any.

uORF proteins	Positions	Epitope	Mutation (HR/MR)	Percentage frequency (%)
**Linear B-cell epitopes**				
1auORF2.ext	22–29	STSRFRPG	R27C	98.99
M.ext	196–202	ASQRVAG		
	213–230	RIGNYKLNTDHSSSSDNI		
ORF3c	14–22	YRYKPHSLS	L21F; K17E	1.58; 1.25
7a.iORF1	34–47	SSGTYEGNSPFHPL		
	74–83	QLRARSVSPK		
N.iORF2	22–32	AVGRDQNNVGP		
ORF9b	5–10	ISEMHP		
	29–39	AVGRDQNNVGP		
	65–69	EDKAF		
**Conformational B-cell epitopes**				
ORF9b (PDB ID:6Z4U)	61–68	LNSLEDKA		

Note that the residue numbers of an epitope (column 2) located in the particular protein (column 1) and its amino acid sequence (column 3) are given along with the (if any) HR/MR (column 4) mutation(s) present in the epitope. Note that column 5 represents the percentage frequency of the HR/MR. For the uORF HR/MR mutations, refer to **Figure 4.**

**Table 2:** Promiscuous CD8+ T-cell epitopes from severe acute respiratory syndrome coronavirus 2 (SARS-CoV-2) unannotated open reading frame (uORF) proteins are listed along with the number of human leukocyte antigen (HLA) I alleles.

uORF proteins	Positions	Epitopes	No. of alleles	CD8+ immunogenicity score	Vaxijen antigenicity score	Population coverage of the alleles	HR/MR mutation	Percentage frequency (%)
ORF3c	28–36	ALHFLLFFR	5	0.27426	0.7293	29.31%	R36I	20.18
6.iORF	5–13	KVSIWNLDY	9	0.29343	0.8195	59.07%		
7a.iORF1	96–104	SPIFLIVAA	6	0.33318	0.6265	33.20%		
	99–107	FLIVAAIVF	7	0.29611	0.6203	24.47%		
	102–110	VAAIVFITL	28	0.43202	0.8679	100.00%		
7b.iORF1	3–11	IIFWFSLEL	17	0.2683	0.8291	99.98%		
M.ext	89–97	ITGGIAIAM	11	0.34671	0.7715	54.64%		
	63–71	KLIFLWLLW	5	0.34287	0.4968	18.61%		
	19–27	GTITVEELK	4	0.29473	1.0976	37.50%		
	151–159	LVIGAVILR	7	0.2601	1.1027	46.67%		
	149–157	SELVIGAVI	11	0.25658	0.6409	43.14%		
ORF9b	41–48	KVYPIILRL	59	0.2634	0.6489	100.00%		
	87–95	LPDEFVVVT	6	0.31977	0.5057	24.2%		

Note that the residue numbers of an epitope (column 2) located in the particular protein (column 1) and its amino acid sequence (column 3) are given along with its immunogenicity (column 5) and antigenicity (column 6) scores. Note that the number of HLA I alleles to which the epitope binds with (column 4) is also given along with the population coverage of the alleles (column 7). Column 8 represents the highly recurring (HR)/moderately recurring (MR) mutation(s) present in the epitope, if any. The percentage frequencies of the HR/MR mutation(s) are given in column 9. For the uORF HR/MR mutations, refer to **Figure 4**.

**Table 3:** Promiscuous CD4+ T-cell epitopes from severe acute respiratory syndrome coronavirus 2 (SARS-CoV-2) unannotated open reading frame (uORF) proteins are listed along with the number of human leukocyte antigen (HLA) II alleles.

uORF proteins	Positions	Epitope	No. of alleles	CD4+ immunogenicity score	Vaxijen antigenicity score	Population coverage of the alleles	HR/MR mutation	Percentage frequency (%)
3a.iORF1 (ORF3c)	2–16	LLLQILFALLQRYRY	2	35.9554	0.6920	14.77%		
	26–40	LLALHFLLFFRALPK	3	38.66696	0.4136	–	R36I	20.18
	4–18	LQILFALLQRYRYKP	2	39.97024	1.1376	14.77%	K17E	1.25
	3–17	LLQILFALLQRYRYK	2	40.33808	0.7138	14.77%	K17E	1.25
M.ext	101–115	VGLMWLSYFIASFRL	2	34.087	0.6658	–		
	55–69	RNRFLYIIKLIFLWL	2	39.4372	0.5114	10.54%		
	187–201	RTLSYYKLGASQRVA	2	39.12528	0.5644	17.55%		
7a.iORF 1	96–110	SPIFLIVAAIVFITL	2	40.0232	0.7118	28.63%		
ORF9b & N.iORF2	42–56	YPIILRLGSPLSLNM	2	38.29864	0.6786	28.79%		
	43–57	PIILRLGSPLSLNMA	2	38.73068	0.7535	28.79%		
	40–54	KVYPIILRLGSPLSL	3	39.42084	0.623	37.64%		
	41–55	VYPIILRLGSPLSLN	2	39.58644	0.5837	28.79%		
S.iORF 2	24–38	YHLMMVFILLPLRSL	3	40.22584	0.8466			
S.iORF1	4–18	GSMLYMSLGPMVLRG	2	38.62404	1.014	17.55%	Y8-, M9-	42.85, 42.97
	3–17	LGSMLYMSLGPMVLR	2	38.86964	1.0898	17.55%	Y8-, M9-	42.85, 42.97
	2–16	LLGSMLYMSLGPMVL	2	39.86928	0.8619	17.55%	Y8-, M9-	42.85, 42.97
	5–19	SMLYMSLGPMVLRGL	2	40.08156	1.0383	17.55%	Y8-, M9-	42.85, 42.97

Note that the residue numbers of an epitope (column 2) located in a particular protein (column 1) and its amino acid sequence (column 3) are given along with its immunogenicity (column 5) and antigenicity (column 6) scores. Note that the number of HLA II alleles to which the epitope bind with (column 4) is also given along with the population coverage of the alleles (column 7). Column 8 represents the HR/MR mutation(s) present in the epitope, if any. The percentage frequencies of the HR/MR mutation(s) are given in column 9. For the uORF HR/MR mutations, refer to **Figure 4**.

### Potent B-Cell Epitopes Present in the Proteins Expressed by uORFs

Among the 23 proteins expressed by the SARS-CoV-2 uORFs, only six of them, namely, 1auORF2.ext, M.ext, ORF3c, 7a.iORF1, N.iORF2, and N.iORF1 (ORF9b), have the B-cell linear epitopes (**Tables 1**, **SD1**). In total, 10 B-cell linear epitopes of ≥5 amino acids length have been identified in the above uORF proteins. Notably, “ASQRVAG” and “RIGNYKLNTDHSSSSDNI” epitopes identified from M.ext have overlap with the epitopes identified from the canonical ORF M protein (current study). The predicted M protein epitopes have also been reported in previous immunoinformatics study (34).

**Figure 3:**
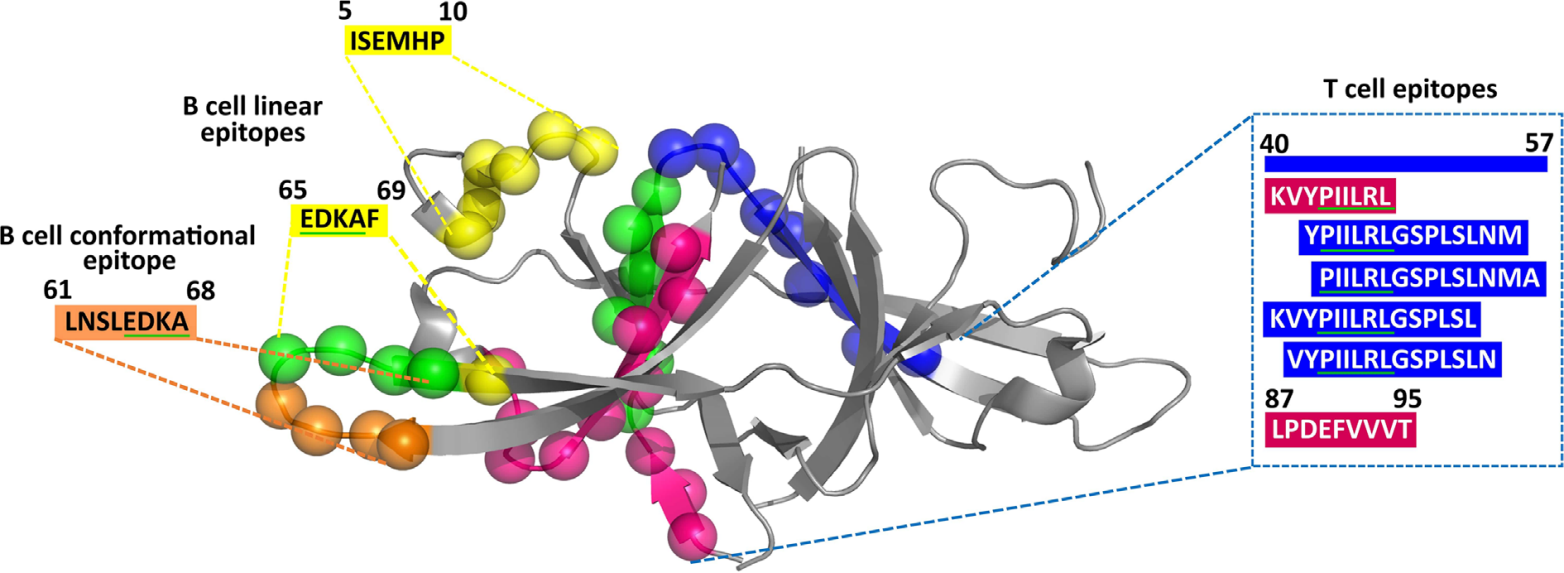
Projection of predicted CD4+ T-cell (residue number: 40–54, 41–55, 42–56, and 43–57) (blue spheres), CD8+ T-cell (residue number: 41–48 and 87–95) (magenta spheres), B-cell conformational (residue number: 61–68) (gold spheres), and B-cell linear (residue number: 5–10 and 65–69) (yellow spheres) epitopes on the crystal structure of ORF9b protein (PDB ID: 6Z4U). Note that the overlapping epitope regions have been indicated in green spheres (in the cartoon representation) and underlined in green (in the amino acid sequence).

Since the structure of ORF9b protein (PDB ID: 6Z4U) alone is known among the uORF proteins, the conformational epitopes present in the ORF9b protein are alone investigated. A recent SARS-CoV-2 immunoglobulin G (IgG) epitope profiling study has shown that along with the spike protein and N protein, ORF9b protein also elicits IgG-specific SARS-CoV-2 response (17). The prediction reveals the presence of one conformational epitope in the ORF9b protein (for the criteria, refer to **Figure 2**), which is of 8 amino acids length (residue numbers 61–68, LNSLEDKA; **Tables 1**, **SD2**). The projection of the predicted epitope onto the protein structure indicates that the epitope is surface exposed (**Figure 3**). It is noteworthy that LNSLEDKA (residue numbers 61–68) conformational epitope has partial overlap with one of the linear B-cell epitopes (EDKAF, residue numbers 65–69; overlapping regions are underlined).

### Potent CD8+ T-Cell Epitopes Present in the uORF Proteins

A total of 340 CD8+ T-cell (HLA I) epitopes (which have percentile rank ≤1) are predicted from 21 uORF proteins [1a.uORF1.ext, 1a.uORF2.ext, S.iORF1, S.iORF2, 3a.iORF1 (ORF3c), 3a.iORF2, M.ext, 6.iORF, 7a.iORF1, 7b.iORF1. N.iORF1 (ORF9b), N.iORF2, 10.iORF, 1a.uORF1, M.iORF, 7a.iORF3, 1a.uORF2, E.iORF, 7b.iORF2, 8.iORF, and 10.uORF] (**Table SD3A**). Among these, 248 epitopes show binding with ≥3 HLA I alleles. Among the 248 epitopes, only 20 have CD8+ immunogenicity score above 0.25 (**Table SD3B**). Refer **Table SD3** for the complete list of CD8+ T-cell epitopes. Finally, 13 CD8+ T-cell epitopes are shortlisted as potent epitopes (**Table 2**) based on the antigenicity and IC_50_ values. Notably, one of the promising ORF9b epitopes, “KVYPIILRL,” has an overlap with the SARS-CoV tor2 strain epitopes (Source: IEDB) (www.iedb.org). Most interestingly, another ORF9b promising epitope (ORF9b_87-95_; “LPDEFVVVT”) predicted here has also been identified in a recent study through IgG epitope profiling (17). 

### Potent CD4+ T-Cell Epitopes Present in uORF Proteins

Fourteen uORF proteins (1a.uORF1.ext, 1a.uORF2.ext, S.iORF1, S.iORF2, 3a.iORF1 (ORF3c), 3a.iORF2, M.ext, 6.iORF, 7a.iORF1, 7b.iORF1, N.iORF1 (ORF9b), N.iORF2, 10.iORF, and 7a.iORF3) are predicted to have 140 CD4+ T-cell epitopes with a percentile rank of ≤1 (**Table SD4A**). Among them, 25 epitopes bind with at least two of the HLA II alleles and have a CD4+ immunogenicity score of ≤40 (**Table SD4B**). By applying the criteria as described in **Figure 2**, 17 epitopes are finally shortlisted as the potent CD4+ T-cell epitopes (**Table 3** **).** Refer **Table SD4C** for the complete list of CD4+ T-cell epitopes

Interestingly, one of the potent ORF9b CD8+ T-cell epitopes, “KVYPIILRL,” has complete overlap with a potent CD4+ T-cell epitope (“KVYPIILRLGSPLSL”; overlapping region is underlined). It also has an overlap with three other ORF9b potent CD4+ T-cell epitopes, namely, “PIILRLGSPLSLNMA,” “YPIILRLGSPLSLNM,” and “VYPIILRLGSPLSLN” (**Figure 3**).

### Population Coverage Exhibited by the Potent CD8+ T-Cell and CD4+ T-Cell Epitopes

The population coverage of the potent CD8+ T-cell and CD4+ T-cell epitopes are subsequently investigated. For this, the 13 CD8+ T-cell and 17 CD4+ T-cell epitopes respectively are tested against the IEDB HLA I and HLA II allele repository. In the case of potent CD8+ T-cell epitopes, the population coverage of the individual epitopes ranges between 18% and 100% (**Tables 2**, **3**). Three of the CD8+ T-cell epitopes have population coverage of about 100%, and one of them is ORF9b epitope “KVYPIILRL.” It further has the highest number of (59 out of 78) HLA I allele-binding partners. Note that population coverage is not simply depicted by the number of alleles that an epitope binds with. Rather, it represents the genotypic frequency of the allele it binds with.

Furthermore, 17 potent CD4+ T-cell epitopes are predicted to have individual population coverage between 10.54% and 37.64%. The ORF9b epitope “KVYPIILRLGSPLSL” has the highest population coverage among the CD4+ T-cell epitopes (*viz.*, 37.64%).

### HLA Class I Supergroup Coverage of Potent CD8+ T-Cell Epitopes

To further investigate the binding specificity or flexibility of the CD8+ T-cell epitopes to HLA class I alleles, the allele-binding coverage of the predicted promiscuous epitopes is analyzed by considering seven HLA class I supergroups (45) and 10 HLA class I supertypes (46). Analysis indicates that only 30% of the promiscuous uORF CD8+ T-cell epitopes fall into the same HLA class I supergroup. For instance, four out of 13 uORF epitopes are specific only to a particular HLA supergroup (**Table SD5**). The rest of the promiscuous epitopes bind to the HLA class I alleles that belong to at least two supergroups.

### Conservation of Epitope Regions

**Figure 4:**
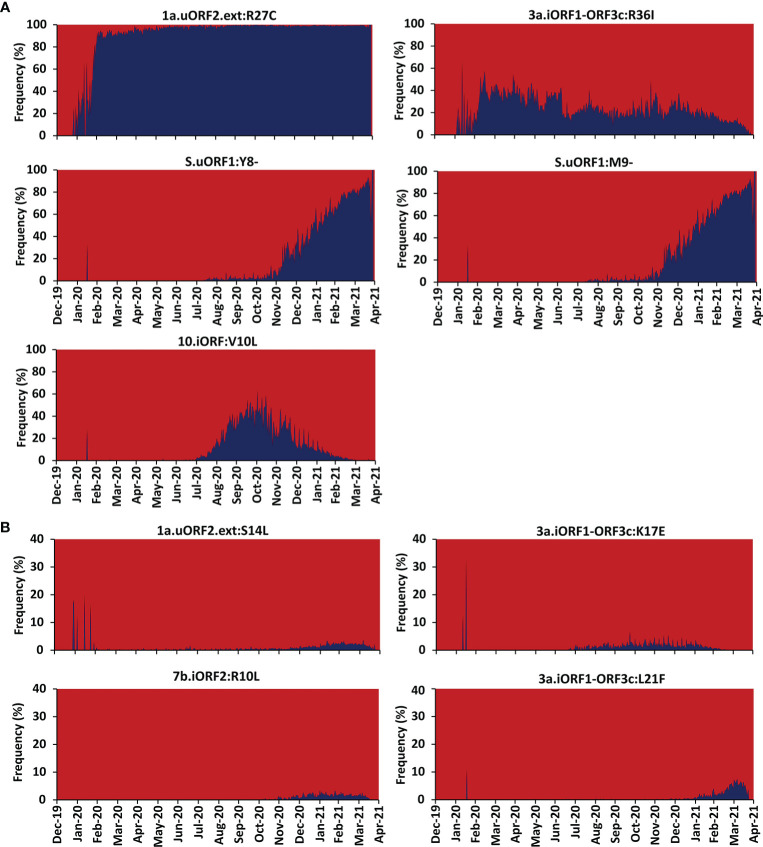
Month-wise occurrence of key recurring severe acute respiratory syndrome coronavirus 2 (SARS-CoV-2) unannotated open reading frame (uORF) mutations. **(A)** Month-wise occurrence of five highly recurring SARS-CoV-2 uORF mutations. Note that the S.uORF1:Y8- and S.uORF1:M9- emerged only after July 2020. **(B)** Month-wise occurrence of four moderately recurring SARS-CoV-2 uORF mutations.

**Figure 5:**
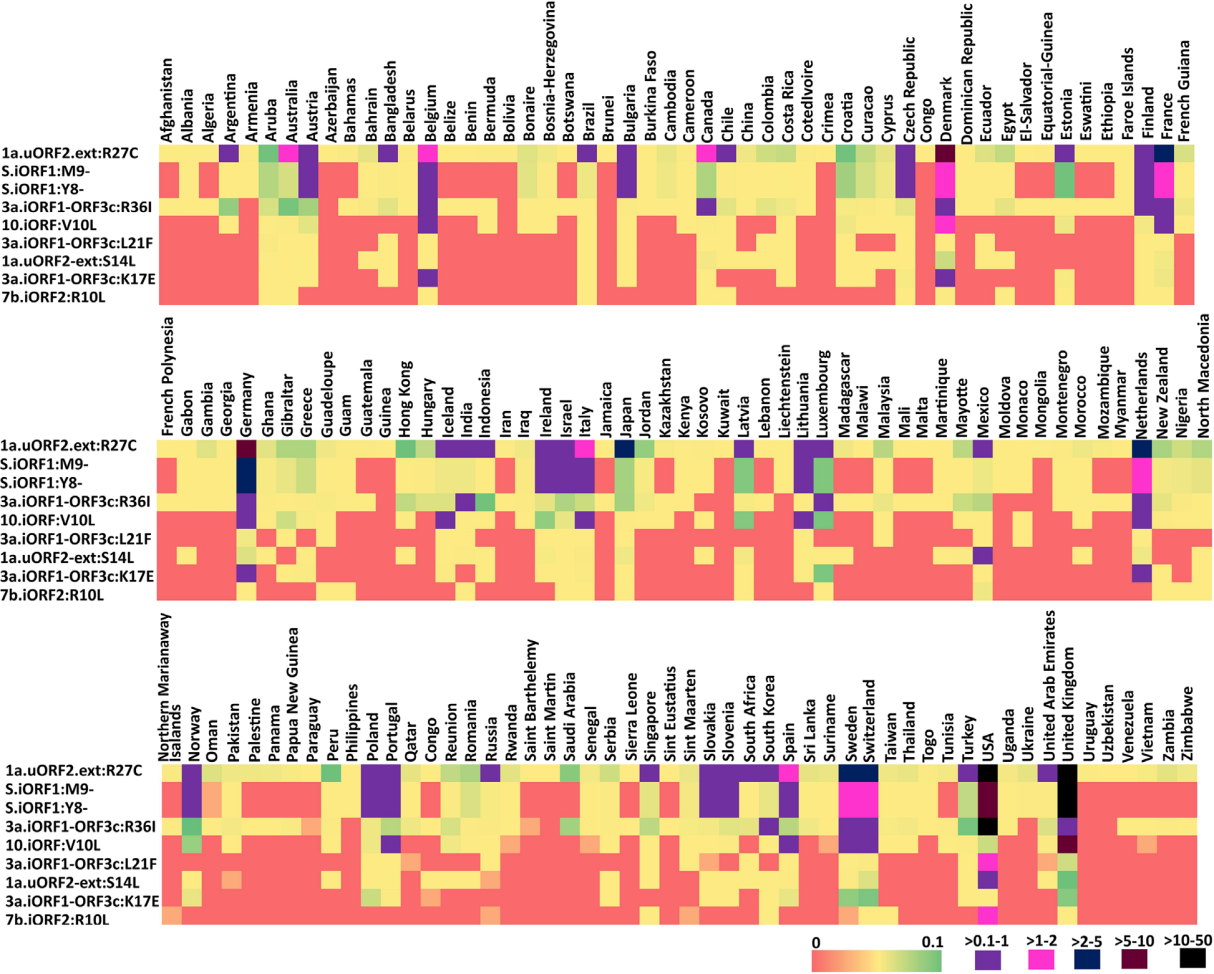
Heat map representing the country-wise percentage frequency of occurrence of five highly recurring (HR) and four moderately recurring (MR) mutations. The first five rows in the heat maps represent the HR mutations, and the last four rows represent the MR mutations.

To investigate the conservation of the predicted SARS-CoV-2 uORF conformational B-cell, linear B-cell, CD8+ T-cell (HLA I), and CD4+ T-cell (HLA II) epitopes, mutational analysis is carried out for the proteins expressed by uORFs. The results reveal that uORF proteins are having 5, 4, and 2,642 high, moderate, and low recurrent mutations, respectively (**Figures 4**, **5**; **Table SD6**). While some of the low-recurring mutations are found in the predicted potential epitope regions, four highly recurring (1a-uORF2-ext: R27C, S.iORF1:Y8-, S.iORF1:M9-, and ORF3c:R36I) and two moderately recurring mutations (ORF3c:L21F and ORF3c:K17E) are found to occur only in 10 out of 41 shortlisted potent epitopes (**Tables 1** **–** **3**). Thus, these epitopes are not considered as potential epitopes.

## Discussion

Even 18 months after the coronavirus disease 2019 (COVID-19) outbreak, treating and preventing SARS-CoV-2 infection are still a big challenge. Especially, the emergence of variants with recurring mutations in the spike protein poses major challenges in treating the SARS-CoV-2 infection. An immunoinformatics approach is employed here to facilitate the multi-epitope vaccine design, which may aid in overcoming the challenges in the traditional vaccine design. Although several studies have been carried out in this regard (26–35), they have mainly focused on predicting the epitopes from the proteins expressed by the canonical ORFs. In addition to the canonical ORF proteins, the uORF proteins of SARS-CoV-2 also exhibit antigenicity (**Table S1**) (17, 51). Nonetheless, there is no systematic investigation carried out to identify the epitopes present in uORF proteins. To this end, the current investigation aims to predict the linear B-cell, conformational B-cell, CD4+ T-cell, and CD8+ T-cell epitopes present in SARS-CoV-2 uORF proteins.

Using the recently published SARS-CoV-2 uORF sequences as the reference sequences (36), the epitopes present in the uORF proteins have been scanned in the IEDB database (www.iedb.org) (50). Based on the cutoff criteria described in **Figure 2**, 10 linear and one conformational B-cell epitopes have been shortlisted as potent epitopes (**Table 1**). Similarly, 17 CD4+ T-cell and 13 CD8+ T-cell epitopes have been shortlisted by considering their antigenicity, immunogenicity, and IC_50_ value (**Tables 2**, **3**). Additionally, the selected CD4+ T-cell and CD8+ T-cell epitopes exhibit an allele coverage of least two (out of 27 HLA II alleles) and three (out of 78 HLA I alleles), respectively. Interestingly, three of the CD8+ T-cell epitopes [“KVYPIILRL” (ORF9b), “IIFWFSLEL” (ORF7b.iORF1), “VAAIVFITL” (ORF7a.iORF1)] exhibit 100% world population coverage, indicating that they are more promising epitopes. Furthermore, CD8+ T-cell epitope “KVYPIILRL” (**Figure 3**) from ORF9b protein exhibits binding with the highest number of HLA I alleles (59 out of 78). This epitope also has an overlapping region with four of the potent CD4+ T-cell epitopes. Thus, these epitopes are the promising T-cell epitopes. Further mutational analyses have confirmed that HR and MR mutations are found only in 10 out of 41 shortlisted potent epitopes (**Tables S1–S3**). Thus, a total of 31 epitopes, *viz.*, nine B-cell and 22 T-cell epitopes are finally shortlisted as potent epitopes from the uORF proteins.

### Potent SARS-CoV-2 Unannotated ORF Epitopes for Multi-Epitope Vaccine Design

From the pool of 31 potent epitopes, 13 epitopes are proposed to be highly suitable for the multi-epitope SARS-CoV-2 vaccine design as discussed below. Since ORF9b followed by M.ext have better expression level among the uORF proteins, epitopes from these proteins can be considered for the vaccine design. The only conformational uORF B-cell epitope [“LNSLEDKA” (ORF9b_61-68_)] and four linear B-cell epitopes [“AVGRDQNNVGP” (ORF9b_29-39,_ N_iORF2_22-32_), “EDKAF” (ORF9b_65-69_), “ASQRVAG” (M.ext_196-202_), and “RIGNYKLNTDHSSSSDNI” (M.ext_213-230_)] can be considered for the vaccine design. However, “EDKAF” (ORF9b_65-69_) is excluded for the vaccine design as it overlaps (underlined) with the conformational B-cell epitope “LNSLEDKA” (ORF9b_61-68_). Thus, a total of four B-cell epitopes are considered for the vaccine design.

**Figure 6:**
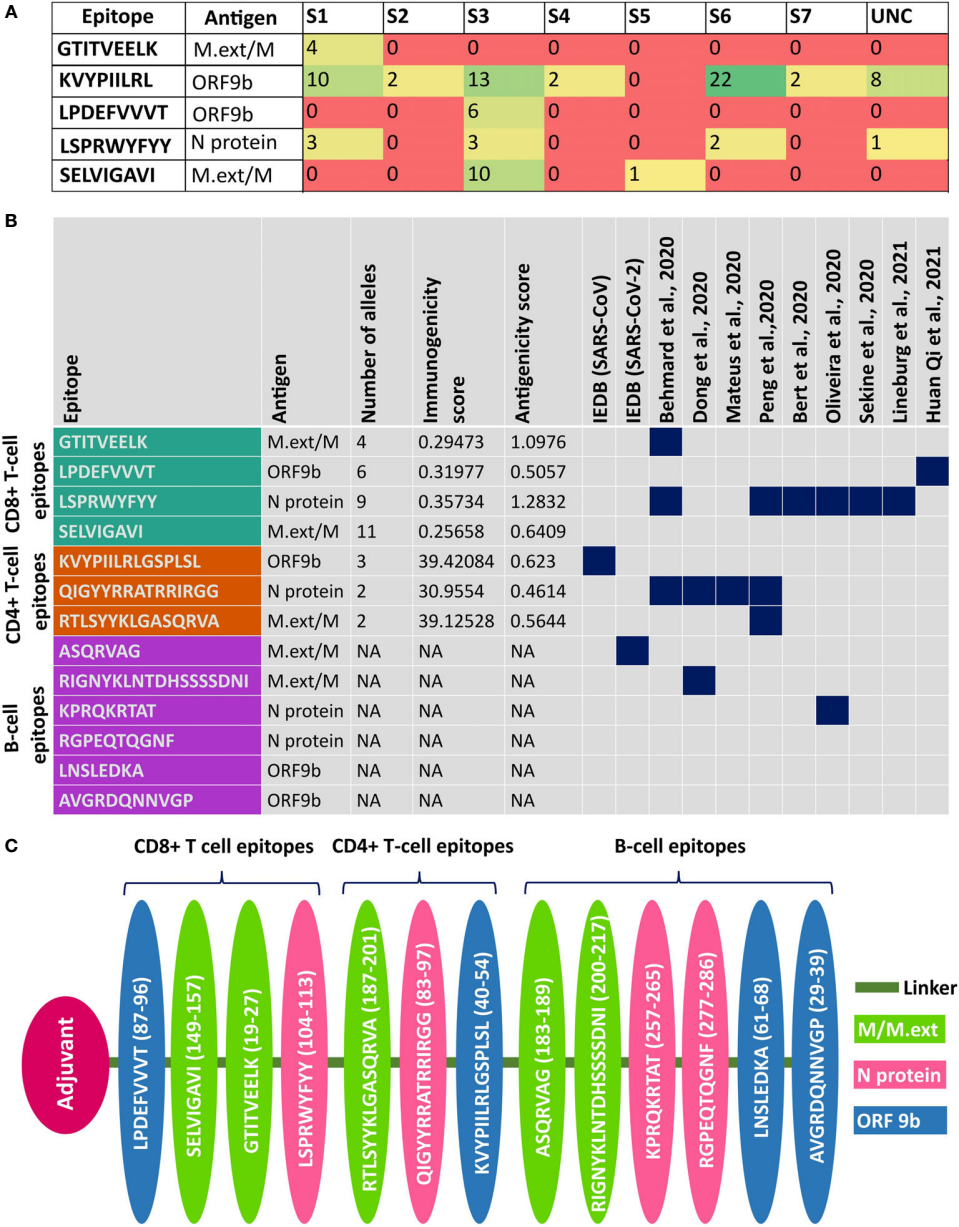
Details about the potent epitopes and the design of multi-epitope vaccine construct. **(A)** Table showing the human leukocyte antigen (HLA) class I supergroup coverage of potent CD8+ T-cell epitopes. **(B)** Summary of the potent CD8+ T-cell, CD4+ T-cell, B-cell linear, and B-cell conformational epitopes used for the vaccine design. Note that if a potent epitope has already been reported elsewhere, it is highlighted in dark blue. **(C)** Linear multi-epitope vaccine construct designed using potent CD8+ T-cell, CD4+ T-cell, and B-cell epitopes predicted from ORF9b, N, and M/M.ext proteins. Note that ORF9b, N, and M/M.ext epitopes are depicted in pink, green, and blue, respectively.

Among the shortlisted potent CD8+ T-cell epitopes, “KVYPIILRL” (ORF9b_40-48_) is predicted to bind with six (S1–S4, S6, and S7) out of seven HLA class I supergroups (**Figure 6A**, **Table SD5**). Interestingly, “SELVIGAVI” (M.ext_149-157_) is the only shortlisted epitope that covers HLA class I supergroup S5 (in addition to S3) that is not covered by “KVYPIILRL” (ORF9b_40-48_); thus, it becomes a valuable candidate for the vaccine design. Although “LPDEFVVVT” (ORF9b_87-95_) (HLA class I supergroup S3) and GTITVEELK (M.ext_19-27_) (HLA class I supergroup S1) cover only one of the HLA class I supergroups, they are also considered for the vaccine design, as they have been reported in earlier investigations (**Figures 6A, B**) (17, 32). In fact, “LPDEFVVVT” (ORF9b_87-95_) is identified to be an epitope of high confidence in a recent IgG profiling experiment (17).

Similarly, the following CD4+ T-cell epitopes can be promising vaccine candidates: “KVYPIILRLGSPLSL” (ORF9b_40-54_), VGLMWLSYFIASFRL” (M.ext_101-115_), and “RTLSYYKLGASQRVA” (M.ext_187-201_ and it overlaps with M.ext_188-202_ CD4+ T-cell epitope). Notably, “KVYPIILRLGSPLSL” (ORF9b_40-54_) overlaps with three other ORF9b CD4+ T-cell epitopes [“YPIILRLGSPLSLNM” (ORF9b_42-56_), “PIILRLGSPLSLNMA” (ORF9b_43-57_), and “VYPIILRLGSPLSLN” (ORF9b_41-55_)]. Furthermore, “KVYPIILRLGSPLSL” (ORF9b_40-54_) overlaps (underlined) with the ORF9b CD8+ T-cell epitope “KVYPIILRL” (ORF9b_40-48_) that covers six out of seven HLA class I supergroups (**Figure 6A** **;** **Tables SD5A, B**). Thus, “KVYPIILRLGSPLSL” (ORF9b_40-54_) is the utmost promising CD4+ T-cell epitope and is considered for the vaccine design. Similarly, “RTLSYYKLGASQRVA” (M.ext_187-201_) is considered to be a potential epitope. Although some of the uORF proteins CD8+ T-cell epitopes have good world population coverage, they are not considered for the vaccine design due to the low expression level of the corresponding protein (36). Such examples include “VAAIVFITL” (7a_iORF1_102-110_, population coverage = 100%) and “IIFWFSLEL” (7b_iORF1_3-11_, population coverage ~100%) epitopes.

Thus, nine epitopes from ORF9b and M.ext proteins are proposed for the multi-epitope SARS-CoV-2 vaccine design (**Figure 6C**). Among the nine uORF epitopes considered for the vaccine design, “KVYPIILRLGSPLSL” (ORF9b_40-54_) is reported in SARS-CoV Tor 2 strain [Source: IEDB] (www.iedb.org). The epitopes “SELVIGAVI” (M.ext_149-157_) (19), GTITVEELK (M.ext_19-28_) (32), “RIGNYKLNTDHSSSSDNI” (M.ext_213-230_) (34), “RTLSYYKLGASQRVA” (M.ext_187-201_) (19), and “LPDEFVVVT” (ORF9b_87-95_) (17) are reported in previous experimental investigations. Since the M.ext protein expressed by the uORF has overlap with the M protein expressed by the corresponding canonical ORF, the M.ext protein epitopes proposed here are also found in M protein.

Due to the high immunodominance and high expression level of canonical ORF proteins, the present study aims to propose a vaccine construct that has the epitopes from both the canonical ORF and uORF proteins. Thus, the potent linear B-cell, conformational B-cell, CD4+ T-cell, and CD8+ T-cell epitopes from canonical ORF proteins have also been investigated independently in this study to propose an efficient multi-epitope-based vaccine construct that encompasses the epitopes from both the canonical ORF and uORF proteins. **Tables SD7–SD10** have the information about the epitopes predicted in the 26 SARS-CoV-2 canonical proteins. By following the same criteria used in the screening and shortlisting of uORF protein epitopes, 41 linear B-cell, five conformational B-cell, 115 CD8+ T-cell, and 71 CD4+ T-cell epitopes are shortlisted as potent epitopes from the canonical ORF proteins (**Tables S3–S6**). Since several epitope prediction studies have been carried out for canonical ORF proteins, the results are not discussed here in detail. The diversity in epitope-HLA class I allele binding is further confirmed by analyzing the epitope binding diversity with respect to different HLA class I supergroups (see *Methods*). In the case of promiscuous epitopes shortlisted from canonical proteins, only 17% of them bind to HLA class I alleles that fall into the same HLA supergroup (**Table SD11**). For instance, 10 out of 71 Nsp1–Nsp16 epitopes and nine out of 44 ORF2 (Spike)–ORF10 epitopes fall into the same HLA I supergroup. Among the shortlisted promising epitopes from the canonical ORF proteins, 36 and 91 have complete and partial overlap (>60%), respectively, with the earlier reported/predicted SARS-CoV-2 epitopes (13–20, 26, 31–35, 52) (**Tables S7A, B**; **Tables SD12, 13**). Thus, the linear B-cell, conformational B-cell, CD8+ T-cell, and CD4+ T-cell epitopes predicted here from the canonical ORF proteins act as a benchmark to validate the prediction of uORF epitopes. Indeed, there is a possibility of excluding the epitopes with good immunogenicity while applying additional criteria like antigenicity and/or binding affinity (IC_50_) (**Figure 2**). However, a detailed comparison between the previously predicted/reported canonical ORF epitopes and the epitopes that are excluded (in the current study) despite having a good immunogenicity score (above 0.25 for CD8+ T-cell and below 40 for CD4+ T-cell epitopes) indicates that only a fraction (<15%) of such epitopes have been excluded (**Tables SD14, 15**).

For the multi-epitope vaccine design, the epitopes predicted from the N protein (encoded by one of the canonical ORFs) are considered, as it tops among the canonical and uORF proteins in terms of the relative translational level (36). “LSPRWYFYY” (N protein_104-112_) and “QIGYYRRATRRIRGG” (N protein_83-97_) are predicted to be potent CD8+ and CD4+ T-cell epitopes, respectively. Notably, the predicted “LSPRWYFYY” (N protein_104-112_) epitope [covers three HLA class I supergroups (S1, S3, and S6)] (**Figure 6A**) overlaps (underlined region) with a previously reported “SPRWYFYYL” immunodominant epitope (**Figure 6B**) (13, 16, 19, 20, 32, 33). Similarly, “QIGYYRRATRRIRGG” (N protein_83-97_) has partial overlap with the previously reported epitope (14, 19, 32, 34). The linear B-cell epitopes “KPRQKRTAT” (N protein_257-265_) and “RGPEQTQGNF” (N protein_277-286_) are considered to be potent epitopes for the vaccine design. Among them, “KPRQKRTAT” (N protein_257-265_) has partial overlap with the conformational B-cell epitope of the N protein and has been reported in previous immunoinformatics studies (33, 34).

Aforementioned potent epitopes identified from N protein (four epitopes), M.ext/M protein (five epitopes), and ORF9b protein (four epitopes) may be more appropriate for the vaccine design, since these proteins occupy the top 3 positions among the SARS-CoV-2 proteins in terms of the relative translational level (as revealed from the ribosome profiling) (36). Considering this point, “SELVIGAVI” (M_138-146,_ M.ext_89-97_), “GTITVEELK” (M_6-14_, M.ext_19-27_), “LSPRWYFYY” (N_104-112_), “LPDEFVVVT” (ORF9b_87-95_), “RTLSYYKLGASQRVA” (M.ext_187-201_, M_174-188_), “KVYPIILRLGSPLSL (ORF9b_40-54_),” “QIGYYRRATRRIRGG” (N_83-97_), “LNSLEDKA” (ORF9b_61-68_), “AVGRDQNNVGP” (ORF9b_29-39_), “ASQRVAG” (M.ext_196-202_, M_183-189_) “RIGNYKLNTDHSSSSDNI” (M.ext_213-230_, M_200-217_), “KPRQKRTAT” (N_257-265_), and “RGPEQTQGNF” (N_277-286_) epitopes predicted in this study can be used for multi-epitope-based SARS-CoV-2 vaccine design, wherein each epitope is connected through a suitable linker region (**Figure 6C**). The multi-epitope vaccine is designed in such a way to cover all the HLA class I supergroups (**Figure 6A**).

Thus, the potent epitopes from the proteins expressed by the canonical ORFs and uORFs of SARS-CoV-2 can be used in the design of multi-epitope vaccine against SARS-CoV-2.

## Conclusions

To facilitate the design of a multi-epitope vaccine against SARS-CoV-2, an immunoinformatics analysis has been carried out here to identify the potential linear B-cell, conformational B-cell, CD4+ T-cell, and CD8+ T-cell epitopes present in the 23 uORF proteins. Using stringent criteria, nine linear B-cell, one conformational B-cell, 17 CD4+ T-cell, and 13 CD8+ T-cell uORF epitopes are shortlisted. Notably, the current study has identified ORF9b epitopes as promising candidates for the multi-epitope vaccine design. “KVYPIILRL” [ORF9b_40-48_] CD8+ T-cell (MHC I/HLA class I) epitope is the most promising epitope not only based on the antigenicity, immunogenicity, and IC50 but also based on its highest HLA class I allele coverage, *viz.*, it covers six out of seven HLA class I supergroups. Furthermore, this region has an overlap with the four potent CD4+ T-cell (MHC II/HLA class II) epitopes. Among the shortlisted uORF epitopes, eight linear B-cell, one conformational B-cell, 10 CD4+ T-cell, and 12 CD8+ T-cell epitopes are finally suggested as potent epitopes based on the mutational analysis. Similar immunoinformatics analysis is also extended for 26 canonical ORF proteins. Considering the high expression level of N protein (encoded by canonical ORF), M/M.ext protein (encoded by canonical/uORF), and ORF9b protein (encoded by uORF), 13 potent epitopes from these proteins are finally considered for the proposed multivalent vaccine design.

## Data Availability Statement

The original contributions presented in the study are included in the article/**Supplementary Material**. Further inquiries can be directed to the corresponding author.

## Author Contributions

PU carried out the immunoinformatics analysis. LP wrote codes for mutation data analysis and plotting. CS wrote scripts to generate the plots and did plotting. LP, CS, and PU analyzed the data. PU and TR wrote the manuscript. PU independently devised the immunoinformatics analysis protocol. TR designed and supervised the project. All authors contributed to the article and approved the submitted version.

## Funding

LP and CS thank MHRD for fellowship. PU thanks CSIR for fellowship.

## Conflict of Interest

The authors declare that the research was conducted in the absence of any commercial or financial relationships that could be construed as a potential conflict of interest.

## Publisher’s Note

All claims expressed in this article are solely those of the authors and do not necessarily represent those of their affiliated organizations, or those of the publisher, the editors and the reviewers. Any product that may be evaluated in this article, or claim that may be made by its manufacturer, is not guaranteed or endorsed by the publisher.
