# Supplementary material for: Revelation of Potent Epitopes Present in Unannotated ORF Antigens of SARS-CoV-2 for Epitope-Based Polyvalent Vaccine Design Using Immunoinformatics Approach
Source: Front Immunol. 2021 Aug 23;12:692937. doi: 10.3389/fimmu.2021.692937 (PMC8419283; doi:10.3389/fimmu.2021.692937)
Supplement: Supplementary file 16 [file Table_1.docx]

# Revelation of potent epitopes present in unannotated ORF antigens of SARS-CoV-2 for epitope-based polyvalent vaccine design using immunoinformatics approach

Patil Pranita Uttamrao, Chakkarai Sathyaseelan^†^, L Ponoop Prasad Patro^†^ and Thenmalarchelvi Rathinavelan*

^†^ These authors contribute equally

*For correspondence: tr@bt.iith.ac.in

**Affiliations**

Department of Biotechnology, Indian Institute of Technology Hyderabad

Kandi Campus, Telangana State -502285, India

**Keywords**

SARS-CoV-2, uORFs, ORF9b, T-cell epitopes, B-cell epitopes, epitope-based polyvalent vaccine, Canonical protein epitopes and HLA I supergroups

**Supplementary Tables**

**Table S1:** Unannotated ORF (uORF) proteins (column 2) listed along with their description (column 3), amino acid lengths (column 4), functions (column 5) and Vaxijen antigenicity scores.

| **S. No** | **Proteins** | **uORF description** | **Size (aa)** | **Function (if known)** | **Vaxijen**  **antigenicity**  **score** |
| --- | --- | --- | --- | --- | --- |
| 1 | 1a.uORF1.ext | extended 1a.uORF1 | 25 |  | 0.92 |
| 2 | 1a.uORF1 | ORF1a uORF 1 | 9 |  | 0.6081 |
| 3 | 1a.uORF2.ext | Extended ORF1a uORF 2 | 33 |  | 0.5148 |
| 4 | 1a.uORF2 | ORF1a uORF 2 | 13 |  | 1.0188 |
| 5 | 1a.iORF | ORF 1a out-of-frame iORF | 5 |  | - |
| 6 | S.iORF1 | extended S.iORF2 | 39 |  | 0.7488 |
| 7 | S.iORF2 | ORF S out-of-frame iORF | 31 |  | 0.8616 |
| 8 | 3a.iORF1 (ORF3c) | ORF 3a out-of-frame iORF | 41 | Predicted transmembrane protein suggestive of viroporin ([*1*](#_heading=h.30j0zll)) | 0.6024 |
| 9 | 3a.iORF2 | ORF 3a out-of-frame iORF | 33 |  | 0.3635 |
| 10 | E.iORF | ORF E iORF | 11 |  | 0.5287 |
| 11 | M.ext | Extended ORF M | 235 |  | 0.4969 |
| 12 | M.iORF | ORF M out-of-frame iORF | 14 |  | 0.6026 |
| 13 | 6.iORF | ORF 6 iORF | 43 |  | 0.4112 |
| 14 | 7a.iORF1 | ORF 7a iORF | 119 |  | 0.6509 |
| 15 | 7a.iORF2 | ORF 7a out-of-frame iORF | 8 |  | 0.7353 |
| 16 | 7a.iORF3 | ORF 7a iORF | 15 |  | 1.8597 |
| 17 | 7b.iORF1 | ORF 7b iORF | 20 |  | 0.8328 |
| 18 | 7b.iORF2 | ORF 7b out-of-frame iORF | 11 |  | 0.0918 |
| 19 | 8.iORF | ORF 8 out-of-frame iORF | 9 |  | 0.3571 |
| 20 | N.iORF1 (ORF9b) | extended N.iORF2 | 97 | Suppress host innate immune response ([*2*](#_heading=h.1fob9te)) | 0.9060 |
| 21 | N.iORF2 | ORF N out-of-frame iORF | 90 |  | 0.8588 |
| 22 | 10.uORF | ORF 10 uORF | 10 |  | 0.8322 |
| 23 | 10.iORF | ORF 10 iORF | 18 |  | 0.9631 |

**Table S2**. List of MHC I (HLA I) and MHC II (HLA II) alleles considered in this study for the CD8+ T-cell and CD4+ T-cell epitope prediction.

| \| HLA class I alleles \| HLA class I alleles \| HLA class I alleles \| HLA class I alleles \| \| --- \| --- \| --- \| --- \| \| 1.A*01:01 \| 21.B*08:01 \| 41.B*48:01 \| 61.C*07:01 \| \| 2.A*02:01 \| 22.B*13:01 \| 42.B*49:01 \| 62.C*07:02 \| \| 3.A*02:06 \| 23.B*13:02 \| 43.B*50:01 \| 63.C*07:04 \| \| 4.A*03:01 \| 24.B*14:02 \| 44.B*51:01 \| 64.C*08:01 \| \| 5.A*11:01 \| 25.B*15:01 \| 45.B*52:01 \| 65.C*08:02 \| \| 6.A*23:01 \| 26.B*15:02 \| 46.B*53:01 \| 66.C*12:02 \| \| 7.A*24:02 \| 27.B*15:25 \| 47.B*55:01 \| 67.C*12:03 \| \| 8.A*25:01 \| 28.B*18:01 \| 48.B*56:01 \| 68.C*14:02 \| \| 9.A*26:01 \| 29.B*27:02 \| 49.B*57:01 \| 69.C*15:02 \| \| 10.A*29:02 \| 30.B*27:05 \| 50.B*58:01 \| 70.C*16:01 \| \| 11.A*30:01 \| 31.B*35:01 \| 51.B*58:02 \| 71.C*17:01 \| \| 12.A*30:02 \| 32.B*35:03 \| 52.C*01:02 \| 72.E*01:01 \| \| 13.A*31:01 \| 33.B*37:01 \| 53.C*02:02 \| 73.E*01:03 \| \| 14.A*32:01 \| 34.B*38:01 \| 54.C*02:09 \| 74.G*01:01 \| \| 15.A*33:01 \| 35.B*39:01 \| 55.C*03:02 \| 75.G*01:02 \| \| 16.A*33:03 \| 36.B*40:01 \| 56.C*03:03 \| 76.G*01:03 \| \| 17.A*68:01 \| 37.B*40:02 \| 57.C*03:04 \| 77.G*01:04 \| \| 18.A*68:02 \| 38.B*44:02 \| 58.C*04:01 \| 78.G*01:06 \| \| 19.A*74:01 \| 39.B*44:03 \| 59.C*05:01 \|  \| \| 20.B*07:02 \| 40.B*46:01 \| 60.C*06:02 \|  \| | \| HLA class II alleles \| HLA class II alleles \| \| --- \| --- \| \| 1.HLA-DRB1*01:01 \| 15.HLA-DQA1*05:01/DQB1*02:01 \| \| 2.HLA-DRB1*03:01 \| 16.HLA-DQA1*05:01/DQB1*03:01 \| \| 3.HLA-DRB1*04:01 \| 17.HLA-DQA1*03:01/DQB1*03:02 \| \| 4.HLA-DRB1*04:05 \| 18.HLA-DQA1*04:01/DQB1*04:02 \| \| 5.HLA-DRB1*07:01 \| 19.HLA-DQA1*01:01/DQB1*05:01 \| \| 6.HLA-DRB1*08:02 \| 20.HLA-DQA1*01:02/DQB1*06:02 \| \| 7.HLA-DRB1*09:01 \| 21.HLA-DPA1*02:01/DPB1*01:01 \| \| 8.HLA-DRB1*11:01 \| 22.HLA-DPA1*01:03/DPB1*02:01 \| \| 9.HLA-DRB1*12:01 \| 23.HLA-DPA1*01:03/DPB1*04:01 \| \| 10.HLA-DRB1*13:02 \| 24.HLA-DPA1*03:01/DPB1*04:02 \| \| 11.HLA-DRB1*15:01 \| 25.HLA-DPA1*02:01/DPB1*05:01 \| \| 12.HLA-DRB3*01:01 \| 26.HLA-DPA1*02:01/DPB1*14:01 \| \| 13.HLA-DRB3*02:02 \| 27.HLA-DRB5*01:01 \| \| 14.HLA-DRB4*01:01 \|  \| |
| --- | --- | --- | --- | --- | --- | --- | --- | --- | --- | --- | --- | --- | --- | --- | --- | --- | --- | --- | --- | --- | --- | --- | --- | --- | --- | --- | --- | --- | --- | --- | --- | --- | --- | --- | --- | --- | --- | --- | --- | --- | --- | --- | --- | --- | --- | --- | --- | --- | --- | --- | --- | --- | --- | --- | --- | --- | --- | --- | --- | --- | --- | --- | --- | --- | --- | --- | --- | --- | --- | --- | --- | --- | --- | --- | --- | --- | --- | --- | --- | --- | --- | --- | --- | --- | --- | --- | --- | --- | --- | --- | --- | --- | --- | --- | --- | --- | --- | --- | --- | --- | --- | --- | --- | --- | --- | --- | --- | --- | --- | --- | --- | --- | --- | --- | --- |

**Table S3.** **Promiscuous B-cell epitopes of SARS-CoV-2 canonical structural proteins (column 1).** Note that the residue numbers of an epitope (column 2) located in a particular protein (column 1) and its amino acid sequence (column 3) are given.

| Linear B-cell epitopes | | |
| --- | --- | --- |
| Proteins | **Position** | **Epitope** |
| E protein | 60-70 | SRVKNLNSSRV |
| M protein | 183-189 | ASQRVAG |
|  | 200-217 | RIGNYKLNTDHSSSSDNI |
| N protein | 5-12 | GPQNQRNA |
|  | 19-47 | GPSDSTGSNQNGERSGARSKQRRPQGLPN |
|  | 61 -66 | KEDLKF |
|  | 72-81 | VPINTNSSPD |
|  | 91-103 | TRRIRGGDGKMKD |
|  | 139-157 | LNTPKDHIGTRNPANNAAI |
|  | 171-213 | FYAEGSRGGSQASSRSSSRSRNSSRNSTPGSSRGTSPARMAGN |
|  | 229-252 | QLESKMSGKGQQQQGQTVTKKSAA |
|  | 257-265 | KPRQKRTAT |
|  | 277-286 | RGPEQTQGNF |
|  | 360-397 | YKTFPPTEPKKDKKKKADETQALPQRQKKQQTVTLLPAA |
|  | 408-415 | QQSMSSAD |
| Spike |  |  |
|  | 15-31 | CVNLTTRTQLPPAYTNS |
|  | 62-73 | VTWFHAIHVSGTNG |
|  | 141-152 | LGVYYHKNNKSW |
|  | 182-186 | KQNGF |
|  | 208-220 | TPINLVRDLPQGF |
|  | 252-259 | GDSSSGWT |
|  | 315-320 | TSNFRV |
|  | 341-361 | VFNATRFASVYAWNRKRISNC |
|  | 384-390 | PTKLNDL |
|  | 405-418 | DEVRQIAPGQTGKI |
|  | 441-448 | LDSKVGGN |
|  | 459-464 | SNLKPF |
|  | 469-478 | STEIYQAGST |
|  | 487-492 | NCYFPL |
|  | 518-523 | LHAPAT |
|  | 525-533 | CGPKKSTNL |
|  | 618-625 | TEVPVAIH |
|  | 657-664 | NNSYECDI |
|  | 674-688 | YQTQTNSPRRARSVA |
|  | 696-709 | TMSLGAENSVAYSN |
|  | 789-798 | YKTPPIKDFG |
|  | 808-813 | DPSKPS |
|  | 1110-1114 | YEPQI |
|  | 1136-1143 | TVYDPLQP |
|  | 1154-1169 | KYFKNHTSPDVDLGDI |
|  | 1256-1163 | FDEDDSEP |
| Conformational B-cell epitopes (linear stretch > 5 amino acids) | | |
| E protein (PDB ID:7K3G) |  | None |
| N protein (NTD) | 59-64 | HGKED |
| N protein (PDB ID-6M3M) | 91-95 | TRRIR |
|  | 101-105 | MKDLS |
| N protein (CTD) |  | None |
| N protein (PDB ID_7C22) | 256-364 | KKPRQKRTATKAYNVTQAFGRRGPEQTQGNFGDQELIRQGTDYKHWPQ  IAQFAPSA SAFFGMSRIGMEVTPSGTWLTYTGAIKLDDKDPNFKDQVILL  NKHIDAYKTFP |
| Spike (PDB ID:6VSB) | 1140-1146 | PLQPELD |

**Table S4.** Promiscuous CD8+ T-cell epitopes (column 3) of SARS-CoV-2 nonstructural proteins (column 4) are listed along with the number of HLA I alleles they bind (column 5) with percentile rank ≤1. Note that the residue numbers of an epitope (column 2) located in a particular protein (column 4) and its amino acid sequence (column 3) are given along with its top (lowest) percentile rank value (predicted from IEDB) (column 6) and the corresponding HLA I allele (column 7). CD8+ T-cell epitopes immunogenicity score (column 8), antigenicity score (column 9) and the binding affinity (IC_50_) with the HLA allele(s) (column 10) are also given.

| S.No | Region | Peptide | Proteins | Number of alleles | Top percentile rank | Top allele(s) | MHC I Immuno  -genicity  score | Vaxijen  Antigenicity score | IC_50_  (nM) |
| --- | --- | --- | --- | --- | --- | --- | --- | --- | --- |
| 1 | 542-560 | LVSDIDITF | Nsp3 | 40 | 0.1 | HLA-C*02:02;HLA-C*02:09;HLA-B*35:01; | 0.2541 | 1.783 | 409.19,409.19,36.74 |
| 2 | 110-118 | HVGEIPVAY | Nsp1 | 29 | 0.01 | HLA-B*15:02; | 0.28861 | 0.6413 | 55.82 |
| 3 | 19-26 | KSVNITFEL | Nsp3 | 29 | 0.01 | HLA-C*15:02; | 0.33033 | 2.1377 | 35.54 |
| 4 | 1455-1463 | STNVTIATY | Nsp3 | 27 | 0.01 | HLA-A*30:02; | 0.25822 | 0.7143 | 30.96 |
| 5 | 284-292 | IVAGGIVAI | Nsp4 | 23 | 0.08 | HLA-A*02:06; | 0.26278 | 0.7674 | 18.76 |
| 6 | 184-192 | FLARGIVFM | Nsp6 | 22 | 0.06 | HLA-A*02:01; | 0.3263 | 0.7022 | 6.13 |
| 7 | 144-152 | DSKEGFFTY | Nsp16 | 22 | 0.02 | HLA-A*25:01; | 0.29543 | 1.03 | 870.13 (231) |
| 8 | 1511-1519 | LAYILFTRF | Nsp3 | 21 | 0.14 | HLA-B*52:01; | 0.29512 | 0.5081 | 1293.18(44) |
| 9 | 91-99 | IAAVITREV | Nsp4 | 20 | 0.01 | HLA-C*12:03; | 0.32256 | 0.7253 | 6.88 |
| 10 | 343-351 | STGYHFREL | Nsp12 | 19 | 0.13 | HLA-C*15:02; | 0.25116 | 1.3083 | 228.06 |
| 11 | 373-381 | VPFWITIAY | Nsp4 | 19 | 0.01 | HLA-B*35:01; | 0.56221 | 1.0982 | 3.31 |
| 12 | 149-157 | YGIATVREV | Nsp13 | 19 | 0.1 | HLA-C*12:03; | 0.26141 | 1.4272 | 31.34 |
| 13 | 1533-1541 | FSYFAVHFI | Nsp3 | 18 | 0.06 | HLA-C*15:02; | 0.28926 | 0.8806 | 25.15 |
| 14 | 177-185 | LSDRVVFVL | Nsp14 | 18 | 0.1 | HLA-C*08:01; | 0.26126 | 0.759 | 175.39 |
| 15 | 151-159 | SALWEIQQV | Nsp8 | 18 | 0.11 | HLA-B*52:01;HLA-C*12:03;HLA-C*15:02; | 0.27663 | 0.7568 | 4455.54,99.91,440.54 |
| 16 | 386-394 | STKHFYWFF | Nsp4 | 18 | 0.12 | HLA-A*32:01; | 0.32841 | 1.341 | 88.3 |
| 17 | 288-296 | KRVDWTIEY | Nsp14 | 17 | 0.01 | HLA-C*07:01;HLA-C*07:02;HLA-B*27:02;HLA-B*27:05; | 0.45878 | 2.4429 | 73.78,86.77 ,1140.86,106.57 |
| 18 | 52-60 | REHEHEIAW | Nsp2 | 17 | 0.01 | HLA-B*44:02;HLA-B*44:03; | 0.37218 | 0.9263 | 38.92,44.87 |
| 19 | 93-101 | AVITREVGF | Nsp4 | 17 | 0.32 | HLA-A*32:01; | 0.28155 | 0.9415 | 1726.84 (253) |
| 20 | 183-191 | MFLARGIVF | Nsp6 | 16 | 0.05 | HLA-C*14:02; | 0.25451 | 0.6099 | 10.84 |
| 21 | 1351-1359 | MPYFFTLLL | Nsp3 | 16 | 0.02 | HLA-B*51:01; | 0.2513 | 0.4915 | 62.51 |
| 22 | 552-560 | TVSWNLREM | Nsp3 | 16 | 0.22 | HLA-C*02:02;HLA-C*02:09; | 0.25463 | 1.8822 | 422.26,422.26 |
| 23 | 245-253 | HYVRITGLY | Nsp13 | 15 | 0.06 | HLA-A*29:02; | 0.25374 | 0.5400 | 30.55 |
| 24 | 30-38 | VVYRAFDIY | Nsp12 | 14 | 0.16 | HLA-A*30:02;HLA-B*15:25; | 0.29566 | 0.7331 | 56.42,24.38 |
| 25 | 453-461 | VSDIDITFL | Nsp3 | 13 | 0.03 | HLA-C*05:01; | 0.38916 | 2.2906 | 23.09 |
| 26 | 17-25 | FLFVAAIFY | Nsp4 | 12 | 0.06 | HLA-A*29:02; | 0.33519 | 0.4421 | 11.35 |
| 27 | 447-455 | VLDWLEEKF | Nsp2 | 12 | 0.12 | HLA-C*05:01; | 0.27204 | 0.6487 | 349.34 |
| 28 | 417-425 | CVEEVTTTL | Nsp3 | 11 | 0.2 | HLA-C*08:02; | 0.26543 | 0.4118 | 490.68 |
| 29 | 289-297 | SHFAIGLAL | Nsp13 | 11 | 0.01 | HLA-B*39:01; | 0.25237 | 1.3872 | 9.06 |
| 30 | 263-271 | FELEDFIPM | Nsp15 | 10 | 0.1 | HLA-B*18:01; | 0.33479 | 1.2669 | 13.87 |
| 31 | 542-550 | KSREETGLL | Nsp2 | 10 | 0.25 | HLA-C*15:02; | 0.27371 | 0.6263 | 453.98 |
| 32 | 418-426 | MAYITGGVV | Nsp2 | 10 | 0.15 | HLA-B*51:01; | 0.25514 | 0.6955 | 228.72 |
| 33 | 912-920 | TSRYWEPEF | Nsp12 | 10 | 0.36 | HLA-C*16:01; | 0.37217 | 0.5982 | 93 |
| 34 | 288-296 | KLNEEIAII | Nsp2 | 10 | 0.03 | HLA-B*13:02; | 0.43221 | 0.6394 | 1241.58 (34.66) |
| 35 | 23-31 | ITFELDERI | Nsp3 | 10 | 0.21 | HLA-B*52:01; | 0.26357 | 1.0232 | 2857.43 (253.09) |
| 36 | 244-252 | EHYVRITGL | Nsp13 | 9 | 0.01 | HLA-B*14:02; | 0.26858 | 0.5647 | 86.19 |
| 37 | 389-397 | HFYWFFSNY | Nsp4 | 9 | 0.04 | HLA-A*29:02; | 0.30249 | 0.6725 | 8.38 |
| 38 | 67-75 | KHADFDTWF | Nsp4 | 9 | 0.03 | HLA-B*38:01; | 0.33208 | 0.4085 | 252.7 |
| 39 | 196-204 | MRNAGIVGV | Nsp12 | 9 | 0.05 | HLA-C*06:02; | 0.25019 | 1.6262 | 92.42 |
| 40 | 808-816 | TLRVEAFEY | Nsp3 | 9 | 0.03 | HLA-B*15:02; | 0.34997 | 0.4509 | 70.9 |
| 41 | 286-294 | FVKRVDWTI | Nsp14 | 9 | 0.54 | HLA-B*13:02; | 0.25278 | 1.9477 | 2047.7 (123.11) |
| 42 | 156-164 | GLPWNVVRI | Nsp14 | 9 | 0.33 | HLA-E*01:01;HLA-E*01:03; | 0.31693 | 0.6233 | 15562.29,15562.29 (390.53) |
| 43 | 229-237 | GYAFEHIVY | Nsp15 | 8 | 0.11 | HLA-A*29:02; | 0.39489 | 0.4935 | 113.6 |
| 44 | 95-103 | ITREVGFVV | Nsp4 | 8 | 0.23 | HLA-C*15:02; | 0.31257 | 0.9122 | 282.53 |
| 45 | 1794-1802 | LVATAEAEL | Nsp3 | 8 | 0.28 | HLA-C*03:03;HLA-C*03:04; | 0.27563 | 0.7661 | 104.4,104.4 |
| 46 | 245-253 | QQWGFTGNL | Nsp14 | 8 | 0.06 | HLA-B*48:01; | 0.28136 | 1.0003 | 423.81 |
| 47 | 360-368 | AYKIEELFY | Nsp14 | 7 | 0.08 | HLA-A*29:02; | 0.31471 | 0.5856 | 161.69 |
| 48 | 444-452 | CPAEIVDTV | Nsp13 | 7 | 0.05 | HLA-B*51:01; | 0.32331 | 0.4414 | 229.6 |
| 49 | 185-193 | GHFAWWTAF | Nsp16 | 7 | 0.17 | HLA-B*38:01; | 0.5572 | 0.6429 | 376.47 |
| 50 | 146-154 | GSVGFNIDY | Nsp5 | 7 | 0.33 | HLA-A*29:02; | 0.28069 | 1.8062 | 199.16 |
| 51 | 286-294 | LLEDEFTPF | Nsp5 | 7 | 0.37 | HLA-B*15:02; | 0.2888 | 0.564 | 242.33 |
| 52 | 147-155 | LSYGIATVR | Nsp13 | 7 | 0.15 | HLA-A*68:01; | 0.25621 | 1.696 | 13.78 |
| 53 | 742-750 | LSLREVRTI | Nsp3 | 7 | 0.22 | HLA-B*52:01; | 0.2512 | 1.5576 | 2272.2 (140.34) |
| 54 | 599-607 | DYGARFYFY | Nsp3 | 6 | 0.27 | HLA-A*29:02; | 0.27625 | 0.5667 | 123.85 |
| 55 | 370-378 | IVVFDEISM | Nsp13 | 6 | 0.57 | HLA-C*03:03;HLA-C*03:04; | 0.26271 | 0.6349 | 241.65,241.65 |
| 56 | 261-269 | SPFELEDFI | Nsp15 | 6 | 0.11 | HLA-B*51:01; | 0.30932 | 0.9378 | 668.87 (135.35) |
| 57 | 54-62 | HEHEIAWYT | Nsp2 | 6 | 0.21 | HLA-B*50:01; | 0.46246 | 0.4819 | 693.26 (125.23) |
| 58 | 285-293 | VAGGIVAIV | Nsp4 | 5 | 0.55 | HLA-B*51:01; | 0.32434 | 0.5099 | 4931.1 |
| 59 | 560-568 | MLAHAEETR | Nsp3 | 4 | 0.65 | HLA-A*33:03; | 0.28478 | 0.588 | 179.56 |
| 60 | 82-90 | NTVIWDYKR | Nsp15 | 4 | 0.08 | HLA-A*33:03;HLA-A*68:01; | 0.25478 | 0.8004 | 16.62,5.92 |
| 61 | 743-751 | SLREVRTIK | Nsp3 | 4 | 0.02 | HLA-A*30:01; | 0.31699 | 0.7073 | 17.43 |
| 62 | 916-924 | WEPEFYEAM | Nsp12 | 4 | 0.16 | HLA-B*18:01; | 0.31503 | 0.525 | 93.5 |
| 63 | 230-238 | YAFEHIVYG | Nsp15 | 4 | 0.39 | HLA-C*12:03; | 0.32821 | 0.8955 | 53.62 |
| 64 | 291-298 | EEIAIILAS | Nsp2 | 3 | 0.29 | HLA-B*18:01; | 0.35095 | 0.6551 | 201.13 |
| 65 | 56-64 | HEIAWYTER | Nsp2 | 3 | 0.49 | HLA-A*33:01; | 0.38605 | 0.5937 | 464.7 |
| 66 | 1889-1897 | HNIALIWNV | Nsp3 | 3 | 0.42 | HLA-A*68:02; | 0.38021 | 0.7397 | 35.67 |
| 67 | 155-163 | KGLPWNVVR | Nsp14 | 3 | 0.05 | HLA-A*31:01; | 0.25381 | 0.6163 | 24.96 |
| 68 | 79-87 | NYQHEETIY | Nsp12 | 3 | 0.24 | HLA-C*14:02; | 0.29722 | 0.5631 | 314.16 |
| 69 | 50-58 | PVNVAFELW | Nsp15 | 3 | 0.35 | HLA-B*58:01; | 0.26576 | 0.8336 | 259.37 |
| 70 | 279-287 | RLIIRENNR | Nsp16 | 3 | 0.14 | HLA-A*31:01; | 0.31253 | 0.6013 | 23.36 |
| 71 | 703-711 | TQLGIEFLK | Nsp3 | 3 | 0.21 | HLA-A*11:01; | 0.34667 | 1.4708 | 31.15 |

**Table S5.** Promiscuous CD8+ T-cell epitopes (column 3) of SARS-CoV-2 structural and accessory proteins (column 4) are listed along with the number of HLA I alleles they bind with percentile rank ≤1 (column 5). Note that the residue numbers of an epitope (column 2) located in a particular protein (column 4) and its amino acid sequence (column 3) are given along with its top (lowest) percentile rank value (predicted from IEDB) (column 6) and the corresponding HLA I allele (column 7). CD8+ T-cell epitopes immunogenicity score (column 8), antigenicity score (column 9) and the binding affinity (IC_50_) with the HLA allele(s) (column 10) are also given.

| S. No | Region | Peptide | Proteins | Number of alleles | Top  percentile  rank | Top allele(s) | MHC I Immuno-genicity  score | Vaxijen  Antigenicity score | IC_50_ (nM) |  |
| --- | --- | --- | --- | --- | --- | --- | --- | --- | --- | --- |
| 1 | 104-112 | VAAIVFITL | ORF7a protein | 28 | 0.04 | HLA-C*03:03;HLA-C*03:04; | 0.43202 | 0.8679 | 31.33 | |
| 2 | 223-231 | TGVEHVTFF | ORF3a | 24 | 0.14 | HLA-C*02:02;HLA-C*02:09; | 0.28567 | 0.6777 | 677 (237.35) | |
| 3 | 5-13 | NVFAFPFTI | ORF10 protein | 18 | 0.04 | HLA-B*52:01;HLA-A*68:02; | 0.30241 | 0.4365 | 230.51,4.36 | |
| 4 | 628-636 | QLTPTWRVY | Spike | 18 | 0.03 | HLA-B*15:02; | 0.31555 | 1.2119 | 87.57 | |
| 5 | 26-34 | IIFWFSLEL | ORF7b protein | 17 | 0.26 | HLA-C*17:01; | 0.2683 | 0.8291 | 180.11 | |
| 6 | 7 -15 | FQVTIAEIL | ORF6 protein | 16 | 0.06 | HLA-B*48:01; | 0.38115 | 0.4445 | 523 | |
| 7 | 7- 15 | IFTIGTVTL | ORF3a protein | 16 | 0.03 | HLA-C*14:02; | 0.27358 | 0.8573 | 9.49 | |
| 8 | 116-124 | QSINFVRII | ORF3a protein | 15 | 0.11 | HLA-B*52:01; | 0.31099 | 0.9407 | 1247.59(64) | |
| 9 | 9-17 | VTIAEILLI | ORF6 protein | 14 | 0.11 | HLA-C*15:02; | 0.28951 | 0.6409 | 102.11 | |
| 10 | 39-47 | ASLPFGWLI | ORF3a protein | 13 | 0.12 | HLA-C*15:02; | 0.3116 | 1.3854 | 110.86 | |
| 11 | 71-79 | IQYIDIGNY | ORF8 protein | 11 | 0.03 | HLA-B*15:01; | 0.30442 | 2.0960 | 32.72 | |
| 12 | 76-84 | ITGGIAIAM | M protein | 11 | 0.51 | HLA-C*03:02; | 0.34671 | 0.7715 | 151.05 | |
| 13 | 136-144 | SELVIGAVI | M protein | 11 | 0.04 | HLA-B*49:01; | 0.25658 | 0.6409 | 83.35 | |
| 14 | 1221-1229 | IAGLIAIVM | Spike | 10 | 0.48 | HLA-C*03:03;HLA-C*03:04; | 0.30271 | 0.4716 | 283.42 | |
| 15 | 5-13 | VFLGIITTV | ORF8 protein | 10 | 0.32 | HLA-A*23:01; | 0.34082 | 0.6983 | 726.84(189) | |
| 16 | 23-31 | KVSIWNLDY | ORF6 protein | 9 | 0.09 | HLA-A*30:02; | 0.29343 | 0.8195 | 26.48 | |
| 17 | 104-112 | LSPRWYFYY | N protein | 9 | 0.21 | HLA-A*29:02; | 0.35734 | 1.2832 | 11.47 | |
| 18 | 62-70 | VTWFHAIHV | Spike | 9 | 0.11 | HLA-C*15:02; | 0.38925 | 0.5426 | 107.78 | |
| 19 | 103-111 | DLSPRWYFY | N protein | 8 | 0.11 | HLA-A*26:01; | 0.25933 | 1.7645 | 663.52(55) | |
| 20 | 227-235 | HVTFFIYNK | ORF3a protein | 7 | 0.08 | HLA-A*68:01; | 0.36278 | 0.9862 | 4.19 | |
| 21 | 138-146 | LVIGAVILR | M protein | 7 | 0.03 | HLA-A*68:01; | 0.2601 | 1.1027 | 8.15 | |
| 22 | 105-113 | SPRWYFYYL | N protein | 7 | 0.07 | HLA-B*07:02; | 0.34101 | 0.734 | 8.33 | |
| 23 | 225-233 | VEHVTFFIY | ORF3a protein | 7 | 0.02 | HLA-B*18:01; | 0.3766 | 0.5216 | 9.45 | |
| 24 | 101-109 | FLIVAAIVF | ORF7a | 7 | 0.25 | HLA-B*46:01; | 0.29611 | 0.6203 | 5688(57.57) | |
| 25 | 7-15 | FAFPFTIYS | ORF10 protein | 6 | 0.29 | HLA-C*12:03; | 0.28754 | 0.8343 | 30.8 | |
| 26 | 229-237 | LPIGINITR | Spike | 6 | 0.32 | HLA-A*33:01; | 0.33581 | 1.8215 | 701.31(98) | |
| 27 | 5-13 | MRIFTIGTV | ORF3a protein | 6 | 0.22 | HLA-C*06:02; | 0.37536 | 0.6991 | 138.72 | |
| 28 | 98-106 | SPIFLIVAA | ORF7a protein | 6 | 0.01 | HLA-B*55:01;HLA-B*56:01; | 0.33318 | 0.6265 | 58.53,20.63 | |
| 29 | 227-235 | VDLPIGINI | Spike | 6 | 0.1 | HLA-B*37:01; | 0.25528 | 1.3808 | 744.85 | |
| 30 | 1209-1217 | YIKWPWYIW | Spike | 6 | 0.16 | HLA-A*32:01; | 0.42524 | 0.9673 | 90.98 | |
| 31 | 58-66 | FFSNVTWFH | Spike | 5 | 0.29 | HLA-A*29:02; | 0.27187 | 0.5951 | 60.84 | |
| 32 | 1089-1097 | FPREGVFVS | Spike | 5 | 0.02 | HLA-B*56:01; | 0.31233 | 0.5509 | 55.4 | |
| 33 | 50-58 | KLIFLWLLW | M protein | 5 | 0.07 | HLA-A*32:01; | 0.34287 | 0.4968 | 29.43 | |
| 34 | 660-668 | YECDIPIGA | Spike | 5 | 0.05 | HLA-B*50:01; | 0.2561 | 0.6385 | 191.81 | |
| 35 | 6-14 | GTITVEELK | M protein | 4 | 0.09 | HLA-A*11:01; | 0.29473 | 1.0976 | 31.09 | |
| 36 | 721-729 | SVTTEILPV | Spike | 4 | 0.24 | HLA-A*02:06; | 0.2586 | 0.8441 | 21.89 | |
| 37 | 827-835 | TLADAGFIK | Spike | 4 | 0.18 | HLA-A*11:01; | 0.28158 | 0.5781 | 40.02 | |
| 38 | 12- 20 | APRITFGGP | N protein | 3 | 0.19 | HLA-B*55:01; | 0.34712 | 0.4775 | 570.66 | |
| 39 | 348-356 | ASVYAWNRK | Spike | 3 | 0.21 | HLA-A*11:01; | 0.28107 | 0.5788 | 29.08 | |
| 40 | 20-28 | FLAFVVFLL | E protein | 3 | 0.27 | HLA-A*02:01; | 0.30188 | 0.5308 | 5.97 | |
| 41 | 118-126 | INFVRIIMR | ORF3a protein | 3 | 0.19 | HLA-A*33:01; | 0.26494 | 0.7646 | 115.28 | |
| 42 | 841-849 | LGDIAARDL | Spike | 3 | 0.22 | HLA-C*08:02; | 0.27269 | 1.6038 | 497.9 | |
| 43 | 1- 9 | MKIILFLAL | ORF7a protein | 3 | 0.38 | HLA-B*14:02; | 0.29002 | 0.4624 | 501.16(95) | |
| 44 | 40-48 | SLPFGWLIV | ORF3a protein | 3 | 0.41 | HLA-C*01:02; | 0.42411 | 1.6587 | 856.65(200) | |

**Table S6.** Promiscuous CD4+ T-cell epitopes (column 3) of SARS-CoV-2 structural, nonstructural and accessory proteins (column 4) are listed along with the number of HLA II alleles they bind with percentile rank ≤1 (column 5). Note that the residue numbers of an epitope (column 2) located in a particular protein (column 4) and its amino acid sequence (column 3) are given along with its lowest percentile rank value (predicted from IEDB) (column 6) and the corresponding HLA II allele (column 7). CD4+ T-cell epitopes immunogenicity score (column 8), antigenicity score (column 9) and binding affinity (IC_50_) with the HLA allele(s) (column 10) are also given.

| S.No | Region | Peptide | Proteins | Number of alleles | Top  Percentile  rank | Top allele(s) | CD4+ Immuno  -genicity  score | Vaxijen  Anti  -genicity score | IC_50_  (nM) |
| --- | --- | --- | --- | --- | --- | --- | --- | --- | --- |
| 1 | 25 | VFLLVTLAILTALRL | E protein | 2 | 0.38 | HLA-DRB1*01:01; | 35.73644 | 0.7218 | 43.63 |
| 2 | 88 | VGLMWLSYFIASFRL | M protein | 3 | 0.07 | HLA-DPA1*01:03/DPB1*02:01; | 34.087 | 0.6658 | 167.5 |
| 3 | 98 | ASFRLFARTRSMWSF | M protein | 2 | 0.48 | HLA-DRB1*11:01; | 25.24444 | 0.7304 | 9.01 |
| 4 | 42 | RNRFLYIIKLIFLWL | M protein | 2 | 0.65 | HLA-DRB1*11:01; | 39.4372 | 0.5114 | 468.61 |
| 5 | 174 | RTLSYYKLGASQRVA | M protein | 2 | 0.06 | HLA-DRB1*09:01; | 39.12528 | 0.5644 | 15.06 |
| 6 | 84 | IGYYRRATRRIRGGD | N protein | 2 | 0.42 | HLA-DRB1*11:01; | 31.83548 | 0.6649 | 23.99 |
| 7 | 83 | QIGYYRRATRRIRGG | N protein | 2 | 0.39 | HLA-DRB1*11:01; | 30.9554 | 0.4614 | 18.24 |
| 8 | 628 | NMLRIMASLVLARKH | Nsp12 | 4 | 0.01 | HLA-DRB1*15:01; | 28.83568 | 0.4897 | 9.7 |
| 9 | 627 | PNMLRIMASLVLARK | Nsp12 | 4 | 0.01 | HLA-DRB1*15:01; | 28.29936 | 0.4128 | 7.68 |
| 10 | 745 | FYAYLRKHFSMMILS | Nsp12 | 3 | 0.44 | HLA-DRB1*11:01; | 39.69504 | 0.4202 | 19.77 |
| 11 | 630 | LRIMASLVLARKHTT | Nsp12 | 3 | 0.32 | HLA-DPA1*02:01/DPB1*14:01; | 27.69396 | 0.6646 | 190.54 |
| 12 | 629 | MLRIMASLVLARKHT | Nsp12 | 3 | 0.2 | HLA-DPA1*02:01/DPB1*14:01; | 28.38828 | 0.5283 | 163.98 |
| 13 | 774 | GLVASIKNFKSVLYY | Nsp12 | 2 | 0.46 | HLA-DRB1*15:01; | 37.31636 | 0.4166 | 12.43 |
| 14 | 542 | MNLKYAISAKNRART | Nsp12 | 2 | 0.08 | HLA-DPA1*02:01/DPB1*14:01; | 30.4728 | 1.4377 | 106.79 |
| 15 | 541 | QMNLKYAISAKNRAR | Nsp12 | 2 | 0.07 | HLA-DPA1*02:01/DPB1*14:01; | 29.1294 | 1.5044 | 88.71 |
| 16 | 540 | TQMNLKYAISAKNRA | Nsp12 | 2 | 0.11 | HLA-DPA1*02:01/DPB1*14:01; | 30.01152 | 1.4529 | 116.67 |
| 17 | 143 | ETFKLSYGIATVREV | Nsp13 | 4 | 0.56 | HLA-DPA1*02:01/DPB1*14:01; | 38.95652 | 0.7612 | 405.54 |
| 18 | 142 | EETFKLSYGIATVRE | Nsp13 | 3 | 0.7 | HLA-DRB1*01:01; | 38.568 | 0.8194 | 5.95 |
| 19 | 141 | TEETFKLSYGIATVR | Nsp13 | 3 | 0.78 | HLA-DRB3*02:02; | 38.79656 | 0.8859 | 36.63 |
| 20 | 182 | FTGYRVTKNSKVQIG | Nsp13 | 2 | 0.27 | HLA-DRB1*07:01; | 35.59132 | 0.7725 | 14.84 |
| 21 | 181 | VFTGYRVTKNSKVQI | Nsp13 | 2 | 0.21 | HLA-DRB1*07:01; | 36.01888 | 0.6256 | 11.3 |
| 22 | 48 | DMTYRRLISMMGFKM | Nsp14 | 5 | 0.41 | HLA-DRB1*04:01; | 31.65644 | 0.6502 | 16.37 |
| 23 | 49 | MTYRRLISMMGFKMN | Nsp14 | 5 | 0.35 | HLA-DPA1*02:01/DPB1*14:01; | 32.279 | 0.5230 | 421 |
| 24 | 50 | TYRRLISMMGFKMNY | Nsp14 | 2 | 0.39 | HLA-DPA1*02:01/DPB1*14:01; | 33.7934 | 0.4691 | 431.19 |
| 25 | 85 | IWDYKRDAPAHISTI | Nsp15 | 2 | 0.21 | HLA-DRB3*01:01; | 39.5272 | 0.5607 | 28.3 |
| 26 | 82 | NTVIWDYKRDAPAHI | Nsp15 | 2 | 0.24 | HLA-DRB3*01:01; | 37.53792 | 0.5217 | 41.27 |
| 27 | 83 | TVIWDYKRDAPAHIS | Nsp15 | 2 | 0.24 | HLA-DRB3*01:01; | 37.01888 | 0.7983 | 34.05 |
| 28 | 84 | VIWDYKRDAPAHIST | Nsp15 | 2 | 0.24 | HLA-DRB3*01:01; | 37.06888 | 0.7102 | 34.98 |
| 29 | 278 | GRLIIRENNRVVISS | Nsp16 | 2 | 0.19 | HLA-DRB1*13:02; | 28.37836 | 0.5903 | 8.01 |
| 30 | 277 | KGRLIIRENNRVVIS | Nsp16 | 2 | 0.18 | HLA-DRB1*13:02; | 29.31836 | 0.7821 | 7.52 |
| 31 | 1394 | EASFNYLKSPNFSKL | Nsp3 | 3 | 0.46 | HLA-DRB1*04:01; | 33.45056 | 0.5954 | 84.79 |
| 32 | 983 | ESPFVMMSAPPAQYE | Nsp3 | 3 | 0.01 | HLA-DRB1*01:01; | 39.49368 | 0.5462 | 3.91 |
| 33 | 1393 | LEASFNYLKSPNFSK | Nsp3 | 3 | 0.61 | HLA-DRB1*04:05; | 34.15716 | 0.6015 | 102.71 |
| 34 | 982 | QESPFVMMSAPPAQY | Nsp3 | 3 | 0.01 | HLA-DRB1*01:01; | 39.24808 | 0.5853 | 3.46 |
| 35 | 984 | SPFVMMSAPPAQYEL | Nsp3 | 3 | 0.01 | HLA-DRB1*01:01; | 39.66152 | 0.5833 | 3.58 |
| 36 | 1591 | CMMCYKRNRATRVEC | Nsp3 | 2 | 0.15 | HLA-DRB3*02:02; | 31.04212 | 1.0731 | 9.53 |
| 37 | 1533 | FSYFAVHFISNSWLM | Nsp3 | 2 | 0.61 | HLA-DPA1*01:03/DPB1*04:01; | 38.5918 | 0.4114 | 50.01 |
| 38 | 235 | KPTVVVNAANVYLKH | Nsp3 | 2 | 0.32 | HLA-DRB1*13:02; | 38.154 | 0.6614 | 9.15 |
| 39 | 1549 | LIINLVQMAPISAMV | Nsp3 | 2 | 0.68 | HLA-DRB1*01:01; | 39.92876 | 0.6837 | 11.46 |
| 40 | 1592 | MMCYKRNRATRVECT | Nsp3 | 2 | 0.11 | HLA-DRB3*02:02; | 35.29056 | 0.9501 | 9.2 |
| 41 | 1589 | STCMMCYKRNRATRV | Nsp3 | 2 | 0.44 | HLA-DRB1*09:01;HLA-DRB3*02:02; | 29.57948 | 0.6671 | 262.85,13.03 |
| 42 | 1534 | SYFAVHFISNSWLMW | Nsp3 | 2 | 0.39 | HLA-DPA1*01:03/DPB1*04:01; | 37.72 | 0.4186 | 38.82 |
| 43 | 1590 | TCMMCYKRNRATRVE | Nsp3 | 2 | 0.15 | HLA-DRB3*02:02; | 29.06016 | 0.8512 | 8.82 |
| 44 | 388 | KHFYWFFSNYLKRRV | Nsp4 | 3 | 0.14 | HLA-DPA1*01:03/DPB1*04:01; | 23.52856 | 0.4111 | 38.38 |
| 45 | 386 | STKHFYWFFSNYLKR | Nsp4 | 2 | 0.25 | HLA-DPA1*01:03/DPB1*04:01; | 28.29724 | 0.4838 | 45.05 |
| 46 | 387 | TKHFYWFFSNYLKRR | Nsp4 | 2 | 0.18 | HLA-DPA1*01:03/DPB1*04:01; | 24.98624 | 0.6182 | 35.16 |
| 47 | 249 | QEFRYMNSQGLLPPK | Nsp6 | 3 | 0.26 | HLA-DRB1*04:05; | 38.01432 | 0.731 | 53.19 |
| 48 | 247 | STQEFRYMNSQGLLP | Nsp6 | 3 | 0.26 | HLA-DRB1*04:05; | 35.52916 | 0.5127 | 42.95 |
| 49 | 248 | TQEFRYMNSQGLLPP | Nsp6 | 3 | 0.26 | HLA-DRB1*04:05; | 36.84096 | 0.5071 | 47.54 |
| 50 | 246 | VSTQEFRYMNSQGLL | Nsp6 | 3 | 0.26 | HLA-DRB1*04:05; | 36.36828 | 0.4972 | 41.74 |
| 51 | 119 | AVVLLILMTARTVYD | Nsp6 | 2 | 0.38 | HLA-DRB1*01:01; | 32.23892 | 0.6962 | 7.47 |
| 52 | 245 | LVSTQEFRYMNSQGL | Nsp6 | 2 | 0.26 | HLA-DRB1*04:05; | 39.09824 | 0.6617 | 63.39 |
| 53 | 164 | MWALIISVTSNYSGV | Nsp6 | 2 | 0.54 | HLA-DRB1*08:02; | 40.37712 | 0.6264 | 143.76 |
| 54 | 118 | SAVVLLILMTARTVY | Nsp6 | 2 | 0.38 | HLA-DRB1*01:01; | 33.18816 | 0.7040 | 10.56 |
| 55 | 120 | VVLLILMTARTVYDD | Nsp6 | 2 | 0.38 | HLA-DRB1*01:01; | 31.25796 | 0.6304 | 6.8 |
| 56 | 165 | WALIISVTSNYSGVV | Nsp6 | 2 | 0.52 | HLA-DRB1*08:02; | 40.42124 | 0.7434 | 148.24 |
| 57 | 183 | PLIVTALRANSAVKL | Nsp8 | 4 | 0.09 | HLA-DRB1*13:02; | 37.38636 | 0.6555 | 8.6 |
| 58 | 184 | LIVTALRANSAVKLQ | Nsp8 | 3 | 0.09 | HLA-DRB1*13:02; | 35.82736 | 0.7473 | 7.22 |
| 59 | 84 | KVKYLYFIKGLNNLN | Nsp9 | 3 | 0.31 | HLA-DRB1*04:05; | 35.8858 | 0.9061 | 69.01 |
| 60 | 86 | KYLYFIKGLNNLNRG | Nsp9 | 2 | 0.3 | HLA-DRB1*04:05; | 35.77856 | 0.6418 | 55.29 |
| 61 | 88 | LYFIKGLNNLNRGMV | Nsp9 | 2 | 0.29 | HLA-DRB1*04:05; | 38.45132 | 0.5197 | 99.17 |
| 62 | 83 | PKVKYLYFIKGLNNL | Nsp9 | 2 | 0.35 | HLA-DPA1*02:01/DPB1*05:01; | 38.9856 | 0.62 | 252.39 |
| 63 | 85 | VKYLYFIKGLNNLNR | Nsp9 | 2 | 0.29 | HLA-DRB1*04:05; | 35.60968 | 0.4595 | 47.43 |
| 64 | 109 | YALVYFLQSINFVRI | orf3aprotein | 3 | 0.15 | HLA-DPA1*01:03/DPB1*04:01; | 40.22592 | 0.7342 | 15.88 |
| 65 | 98 | SPIFLIVAAIVFITL | ORF7a protein | 3 | 0.4 | HLA-DRB1*07:01; | 40.0232 | 0.7118 | 539.2 |
| 66 | 13 | FLAFLLFLVLIMLII | ORF7b protein | 5 | 0.06 | HLA-DPA1*03:01/DPB1*04:02; | 39.99392 | 0.4966 | 534.82 |
| 67 | 43 | SKWYIRVGARKSAPL | ORF8 protein | 2 | 0.72 | HLA-DRB1*11:01; | 38.60556 | 0.8829 | 18.56 |
| 68 | 113 | KTQSLLIVNNATNVV | Spike | 3 | 0.01 | HLA-DRB1*13:02; | 38.7404 | 0.6303 | 6.37 |
| 69 | 115 | QSLLIVNNATNVVIK | Spike | 3 | 0.01 | HLA-DRB1*13:02; | 39.69544 | 0.4343 | 4.56 |
| 70 | 114 | TQSLLIVNNATNVVI | Spike | 3 | 0.01 | HLA-DRB1*13:02; | 38.64296 | 0.4333 | 5.16 |
| 71 | 237 | RFQTLLALHRSYLTP | Spike | 2 | 0.58 | HLA-DRB5*01:01; | 37.39948 | 0.5470 | 10.27 |

**Table S7 (a).** Statistics describing the comparison between the promiscuous canonical ORF epitopes predicted in the current study and the previously predicted/reported SARS-CoV/SARS-CoV-2 epitopes. Note that a predicted epitope is considered to have a partial overlap with the reference epitope if there is a match of 6 and 9 amino acids respectively in the cases of CD8+ T-cell (9-mer ([*3*](#_heading=h.3znysh7))) and CD4+ T-cell (15-mer ([*3*](#_heading=h.3znysh7))) epitopes due to the difference in their lengths. ‘*’ indicates the total number of epitopes that are found either in SARS-CoV, SARS-CoV-2 or both.

| Type of epitope | Number of promiscuous epitopes | Complete overlap | | | | Partial overlap | | | |
| --- | --- | --- | --- | --- | --- | --- | --- | --- | --- |
|  |  | IEDB | | | Published SARS-  CoV-2 epitope | IEDB | | | Published SARS-  CoV-2 epitope |
|  |  | SARS-CoV | SARS-CoV-2 | Total* |  | SARS-CoV | SARS-CoV-2 | Total* |  |
| CD8+ T-cell | 115 | 26 | 7 | 30 | 23 | 52 | 12 | 60 | 36 |
| CD4+ T-cell | 71 | 6 | 2 | 8 | 10 | 46 | 7 | 51 | 38 |
| Linear B-cell | 41 | 4 | 2 | 6 | 2 | 17 | 4 | 20 | 15 |
| Conformational  B-cell | 5 | 1 | 1 | 2 | 1 | 2 | 2 | 3 | 1 |

**Table S7 (b).** Statistics describing the comparison between the promiscuous unannotated ORF epitopes predicted in the current study and the previously predicted/reported SARS-CoV/SARS-CoV-2 epitopes. Note that a predicted epitope is considered to have a partial overlap with the reference epitope if there is a match of 6 and 9 amino acids respectively in the cases of CD8+ T-cell (9-mer ([*3*](#_heading=h.3znysh7))) and CD4+ T-cell (15-mer ([*3*](#_heading=h.3znysh7))) epitopes due to the difference in their lengths. ‘*’ indicates the total number of epitopes that are found either in SARS-CoV, SARS-CoV-2 or both.

| Type of epitope | Number of promiscuous  epitopes | Complete overlap | | | | Partial overlap | | | |
| --- | --- | --- | --- | --- | --- | --- | --- | --- | --- |
|  |  | IEDB | | | Published SARS-  CoV-2 epitope | IEDB | | | Published SARS-  CoV-2 epitope |
|  |  | SARS-CoV | SARS-CoV-2 | Total* |  | SARS-CoV | SARS-CoV-2 | Total* |  |
| CD8+ T-cell | 13 | 2 | 0 | 2 | 5 | 7 | 2 | 8 | 6 |
| CD4+ T-cell | 17 | 0 | 0 | 0 | 1 | 4 | 2 | 5 | 1 |
| Linear B-cell | 10 | 0 | 2 | 2 | 0 | 4 | 2 | 5 | 1 |
| Conformational B-cell | 1 | 0 | 0 | 0 | 0 | 1 | 0 | 1 | 0 |

**References**

1. A. E. Firth, A putative new SARS-CoV protein, 3c, encoded in an ORF overlapping ORF3a. *The Journal of general virology* **101**, 1085-1089 (2020); published online EpubOct (10.1099/jgv.0.001469).

2. H. W. Jiang, H. N. Zhang, Q. F. Meng, J. Xie, Y. Li, H. Chen, Y. X. Zheng, X. N. Wang, H. Qi, J. Zhang, P. H. Wang, Z. G. Han, S. C. Tao, SARS-CoV-2 Orf9b suppresses type I interferon responses by targeting TOM70. *Cellular & molecular immunology* **17**, 998-1000 (2020); published online EpubSep (10.1038/s41423-020-0514-8).

3. W. Fleri, S. Paul, S. K. Dhanda, S. Mahajan, X. Xu, B. Peters, A. Sette, The Immune Epitope Database and Analysis Resource in Epitope Discovery and Synthetic Vaccine Design. *Frontiers in immunology* **8**, 278 (2017)10.3389/fimmu.2017.00278).
